# Recommendation on instrument-based screening for depression during pregnancy and the postpartum period

**DOI:** 10.1503/cmaj.220290

**Published:** 2022-07-25

**Authors:** Eddy Lang, Heather Colquhoun, John C. LeBlanc, John J. Riva, Ainsley Moore, Gregory Traversy, Brenda Wilson, Roland Grad

**Affiliations:** Department of Emergency Medicine (Lang), University of Calgary; Alberta Health Services (Lang), Calgary, Alta.; Department of Occupational Science and Occupational Therapy (Colquhoun), University of Toronto, Toronto, Ont.; Department of Pediatrics (LeBlanc), Dalhousie University, Halifax, NS.; Department of Family Medicine (Riva, Moore), McMaster University, Hamilton, Ont.; Public Health Agency of Canada (Traversy), Ottawa, Ont.; Division of Community Health and Humanities (Wilson), Memorial University, St. John’s, Nfld.; Department of Family Medicine (Grad), McGill University, Montréal, Que.

**KEY POINTS**
- The Canadian Task Force on Preventive Health Care recommends against screening all individuals for depression during pregnancy and the postpartum period (up to 1 year after childbirth), using questionnaires (conditional recommendation, very low–certainty evidence).
- Screening involves using an instrument such as a questionnaire for all patients in a particular setting (e.g., a clinic) whether or not they have symptoms, and using a cut-off score as a threshold to determine who needs to be further evaluated.
- The evidence identified by the task force was very uncertain as to whether screening confers benefit above usual clinical care.
- This recommendation assumes that usual care during pregnancy and the postpartum period includes inquiry and attention to mental health and well-being.

*Key messages for the public*

- Depression during pregnancy or the first year after childbirth is a serious health concern, and there are effective treatments.
- It is very uncertain whether the routine use of a questionnaire for depression administered to all patients in a clinic or other setting improves outcomes.
- As part of usual care, your health care provider should ask you about your well-being and may also ask you about depression symptoms.
- Symptoms such as sad mood, difficulty enjoying activities that you usually like to do, feelings of worthlessness or guilt, unexpected fatigue or lack of energy, or unexpected changes in your sleep patterns are important to discuss with your health care provider.
- If you receive a diagnosis of depression, your health care provider can discuss options for support and treatment.

Depression during pregnancy and the postpartum period can have far-reaching impacts on the child-bearing individual and their infant, as well as on parent–infant interactions and relationships with partners.^1^ Consequences for the child-bearing individual include increased likelihood of future anxiety or depression, lower quality of life, increases in risky behaviours (e.g., tobacco smoking or alcohol consumption) and suicidal ideation.^1^ Impacts on the infant could include delays in physical and mental development, such as cognitive and language development, and overall infant health concerns.^1^ Impacts on parent–infant interactions can include reduced breastfeeding and poor parent–infant bonding.^1^,^2^

Diagnostic criteria for major depressive disorder require patients to have at least 5 symptoms, including depressed mood or diminished interest in activities, and to be experiencing significant distress or functional impairment nearly every day for at least 2 weeks.^3^ Symptoms may include significant weight or appetite change, insomnia or hypersomnia, psychomotor agitation or retardation, fatigue or loss of energy, feelings of worthlessness or guilt, reduced concentration or indecisiveness, and thoughts of death or suicidal ideation.^3^

A 2005 systematic review estimated that the point prevalence of major depression during pregnancy and postpartum ranges from 1% to 6% at different time points (from the first trimester of pregnancy to 1 year postpartum), based on 2–6 studies at any given time point (*n* = 111 to 2104 participants).^4^ A 2008 national survey from the United States of more than 14 000 participants aged 18–50 years reported that the 12-month period prevalence of depression was 8% among pregnant individuals and 9% in postpartum individuals, compared with 8% among nonpregnant individuals.^5^

Usual clinical care during pregnancy and the postpartum period should include discussion with pregnant or postpartum people about their history of mental illness, current symptoms and well-being.^6^ In addition to usual clinical care, screening for depression involves the systematic administration of a screening instrument (most commonly a questionnaire or small set of questions), with a predefined cut-off score, to all pregnant or postpartum people in a particular setting such as a clinic.^7^,^8^ Individuals who meet or exceed the cut-off score are considered “screen positive” and are then further evaluated to see whether they meet diagnostic criteria for depression, whereas those below the cut-off score are “screen negative” and are not usually subject to further evaluation.^8^,^9^ The goal of screening is to identify and help individuals who, without a screening protocol, would have been identified later in their illness or not at all.

Common depression screening instruments include the Patient Health Questionnaire (PHQ) and the Edinburgh Postnatal Depression Scale (EPDS) for postpartum and pregnant individuals. 8,10,11 Depression symptom questionnaires can also be used for several purposes other than screening, including as part of a diagnostic assessment of people suspected of having depression, to track treatment progress or to check for relapse among people with a history of depression.^7^

We draw an important distinction between standard questions posed in a systematic screening context and those integrated into clinical enquiry, based on how a practitioner makes a judgment about next steps. In systematic sceening, all patients meeting the cut-off score are considered “screen positive” and investigated further with diagnostic approaches. In contrast, making a judgment about a patient’s status after a personalized assessment, based on all information available to a practitioner, is considered to be routine clinical care and not screening. For example, if a provider were to ask a patient questions similar to those included in a screening instrument such as the PHQ (e.g., “over the last 2 weeks, how often have you been bothered by little interest or pleasure in doing things; feeling down, depressed, or hopeless?”), and then proceed using their clinical judgment, based on the patient’s responses along with other information about the patient, this would not be considered screening. Rather, it is a flexible and personalized approach, in contrast to the systematic and structured approach of screening using an instrument with a cut-off score. Conversely, if a clinician applied the PHQ questions to all pregnant and postpartum patients in the practice and had a predetermined action based on the responses, then this would constitute screening. Once clinicians suspect depression and begin to investigate it, they are engaging in a diagnostic process whether or not they used a formal depression screening tool.

In 2013, the Canadian Task Force on Preventive Health Care recommended against screening for depression among perinatal or postpartum individuals.^12^ Because use of such screening continues to vary in Canada, updated guidance, based on a review of benefits and harms of screening and taking into account patient preferences, will provide clarity for providers. This guideline replaces the recommendation for pregnant and postpartum individuals from the 2013 guideline.

## Scope

This recommendation provides guidance to primary care health professionals (e.g., physicians, nurses, midwives or other providers who could serve as first point of contact for care during pregnancy or the postpartum period), policy-makers and patients on screening (as defined above) for depression in individuals during pregnancy and up to 1 year postpartum.^13^ The scope of this recommendation also extends to individuals who may be at an elevated risk of depression (e.g., trauma in early life, family history of depression^14^). This recommendation does not extend to individuals with a personal history or current diagnosis of depression or another mental health disorder, those currently receiving assessment or treatment for mental health disorders, those receiving care in psychiatric or other mental health settings, or those who are seeking services owing to symptoms of depression.

This recommendation does not apply to usual care where the provider asks questions about and discusses a patient’s mental health and proceeds based on their clinical judgment; nor does it apply to diagnostic pathways where the clinician suspects that the individual may have depression and tests them accordingly. This guideline does not address depression treatment.

## Recommendation

*The Canadian Task Force on Preventive Health Care recommends against instrument-based depression screening using a questionnaire with cut-off score to distinguish “screen positive” and “screen negative” administered to all individuals during pregnancy and the postpartum period (up to 1 year after childbirth) (conditional recommendation, very low–certainty evidence).*

This recommendation assumes that, as part of usual care during pregnancy and the postpartum period, care providers will inquire about and be attentive to mental health and well-being.

Grading of recommendations is described in Box 1. A summary of the recommendation is available in Box 2.

Box 1: **Grading of recommendations**

Recommendations are graded according to the Grading of Recommendations Assessment, Development and Evaluation (GRADE) system.^15^ Whether a recommendation is strong or conditional will depend on considerations such as certainty in estimated effects of an intervention, including magnitude, as well as estimates of how patients value and prioritize outcomes, variability of these estimates and wise use of resources.

**Strong recommendations**

- Strong recommendations are those for which the Canadian Task Force on Preventive Health Care is confident that the desirable effects of an intervention outweigh its undesirable effects (strong recommendation for an intervention) or that the undesirable effects of an intervention outweigh its desirable effects (strong recommendation against an intervention). A strong recommendation implies that most people will be best served by the recommended course of action.
- Strong recommendations are typically based on high-certainty evidence (i.e., high confidence in the estimate of the effect of an intervention). Strong recommendations may recommend in favour of an intervention (when there is high confidence of net benefit) or against an intervention (when there is high confidence of net harm). However, there are circumstances in which a strong recommendation may be considered based on low- or very low–certainty evidence or when there is absence of evidence or low-certainty evidence of benefit.^16^
- When there is an absence of evidence to provide confidence that there is benefit from implementing a new prevention service or when a conclusion of possible benefit requires a high level of speculation on linkages of uncertain evidence, but there is high certainty that some patients would be harmed or scarce health care resources expended, the task force may make a strong recommendation against service implementation.^17^ This is consistent with the GRADE approach, in which strong recommendations are sometimes made with low-certainty evidence combined with high certainty of harm or resource implications, and with the value that the task force places on using scarce primary care resources wisely.^17^

**Conditional recommendations**

- Conditional recommendations are those for which the desirable effects probably outweigh the undesirable effects (conditional recommendation in favour of an intervention) or undesirable effects probably outweigh the desirable effects (conditional recommendation against an intervention) but appreciable uncertainty exists. Conditional recommendations are made when the certainty of evidence is lower, when the margin between desirable and undesirable consequences is small and the balance depends on patient values and preferences, or when there is high variability in the values and preferences of patients. Conditional recommendations may also be applied when the balance of cost and benefits is ambiguous, key stakeholders differ about the acceptability or feasibility of the implementation, or the effects on health equity are unclear.
- In certain cases where a conditional recommendation for an intervention is made, clinicians are encouraged to engage in shared decision-making, to recognize that different choices will be appropriate for individual patients and to help each person arrive at a management decision consistent with their values and preferences. Clinicians should recognize that different choices will be appropriate for different patients and that decisions must be consistent with each patient’s values and preferences. Knowledge translation tools are available on the task force website (www.canadiantaskforce.ca) to facilitate decisions that are evidence informed and aligned with an individual’s priorities.
- Evidence is graded as high-, moderate-, low- or very low–certainty, based on how likely further research is to change the task force’s confidence in the estimate of effect.

Box 2: **Summary of recommendation for clinicians and policy-makers**

*The Canadian Task Force on Preventive Health Care recommends against instrument-based depression screening using a questionnaire with cut-off score to distinguish “screen positive” and “screen negative” administered to all individuals during pregnancy and the postpartum period (up to 1 year after childbirth) (conditional recommendation, very low–certainty evidence).*

This recommendation does not apply to pregnant or postpartum individuals with personal history of depression, or those already receiving assessment or treatment for other mental disorders.

This recommendation refers to a process of primary care providers administering a depression screening instrument such as a questionnaire in every patient not reporting symptoms of depression, and using a pre-identified cut-off score to classify patients as having positive or negative screening results. When caring for individuals in the pregnant and postpartum period, clinicians should continue to ask about mood or other symptoms of depression, maintain a high degree of vigilance for symptoms and signs of depression, and investigate accordingly.

We found 1 randomized controlled trial (RCT) that evaluated systematic depression screening among postpartum individuals (described as “mothers” or “women” in the study) in Hong Kong (*n* = 462).^18^,^19^ Participants were randomly assigned to receive screening with the EPDS by nurses (*n* = 231) or no such screening (*n* = 231) at 2 months postpartum.^18^ Both groups received usual clinical care, including inquiring about feelings, appetite, sleep, child care and suicidal ideation.^18^ All participants identified as potentially depressed (based on EPDS score ≥ 10 or clinical assessment in the intervention group, or based on clinical assessment alone in the control group) were to be offered counselling or management by a community psychiatric team.^18^ Outcomes were assessed at 6 months postpartum (i.e., 4 months after randomization).^18^

At baseline (2 mo postnatal), 67 participants (29.0%) were identified as potentially depressed in the intervention group and 14 (6.1%) in the control group.^18^ However, we found the effect of screening at 6 months postnatal to be very uncertain in this study for both the critical and important outcomes of interest we selected for this guideline (see Methods section).^19^

The critical outcomes we evaluated included the number depressed based on an EPDS score of 10 or higher (91 fewer per 1000, 95% confidence interval [CI] 24 fewer to 135 fewer) and depression symptoms based on patients’ continuous EPDS scores (0–30, with higher indicating worse; mean difference [MD] 1.36 lower, 95% CI 0.63 lower to 2.09 lower). The critical outcomes also included general mental health symptoms based on the General Health Questionnnaire–12 score (0–36, with higher indicating worse condition; MD 0.33 lower, 95% CI 0.70 lower to 0.04 higher), and their reported or observed capacity to parent (Parenting Stress Index [PSI] total score 0–180, with higher indicating worse; MD 2.78 lower, 95% CI 5.74 lower to 0.18 higher), and PSI Parental Distress subscale (0–60, with higher being worse; MD 1.21 lower, 95% CI 2.48 lower to 0.06 higher).^19^

We also found the effect of screening in this study to be very uncertain for the important outcomes of interest. These included parent–child stress (based on the PSI Parent–Child Dysfunctional Interaction and PSI Difficult Child subscales), marital stress (based on the Kansas Marital Satisfaction Scale) and the number of infant hospital admissions.^19^

The effects of screening on all of these outcomes (critical and important) in this study were very uncertain, owing to very serious risk-of-bias concerns (use of self-reported outcome measures and selective outcome reporting) as well as imprecision concerns because there was only a single small trial.^19^ This very low certainty means that the true effects of screening are likely substantially different from the study data above.^15^ Additionally, in this study, there was little to no difference in mean infant body weight at 6 months comparing screening to no screening (low-certainty evidence).^19^ No adverse events were noted (very low–certainty evidence).^18^ We did not identify any trials evaluating other outcomes of interest (i.e., suicidality, false-positive screens, overdiagnosis, overtreatment, or labelling or stigma).^19^ We also did not find any trials that compared questionnaire-based depression screening to no screening during pregnancy.

### Patient values and preferences

We assessed patient values and preferences for screening during patient engagement activities conducted for this guideline, 20,21 as described in the Methods section. Participants expressed concerns that they might not recognize their own symptoms of depression or take the initiative to seek input from their primary care provider and stated a preference for being screened. However, we noted that “while participants rated their preference to be screened fairly highly in the survey, focus group discussions indicated that participants felt most strongly about having a discussion with a health care provider about their mental health and well-being, rather than a formal screening process. They felt a discussion about depression with a primary health care provider during the pregnancy and [postpartum] period is critical.”^21^ Thus, patient engagement suggested that discussions with health care providers about depression are important to patients.

### Resource use

The task force did not systematically review the resource use or cost-effectiveness of depression screening. In the judgment of the task force, the resource implications of a recommendation against screening are unknown. It is possible that resource savings (e.g., primary care provider time, unnecessary follow-up and cost of inappropriate treatment) would occur in primary care settings that had previously administered screening instruments to all individuals during pregnancy and the postpartum period. However, implications may vary depending on practice setting and are not clear.

### Feasibility, acceptability and equity

In the judgment of the task force, a recommendation against screening is feasible. Primary care providers are trained in recognizing the signs and symptoms of depression during pregnancy and the postpartum period, as well as processes for assessment, treatment and referral (as required) as part of usual clinical care. The extent to which primary care providers are using questionnaire-based screening across Canada as part of usual clinical care is unknown.

Consistent with patient values and preferences that emphasize the desire to have discussions with health care providers, the recommendation against screening for depression using questionnaires administered to all pregnant and postpartum people should be acceptable to most patients, as long as providers continue to inquire about mental health and well-being as part of usual care. In the judgment of the task force, supporting the practice of discussions regarding mental health and well-being within the context of usual clinical care is consistent with a recommendation against screening. The task force considers this recommendation would be acceptable to some stakeholders, such as primary care providers and policy-makers, as it highlights the lack of evidence to support screening but supports the clinical practice of inquiring about mental health.

The task force recognizes that a recommendation against screening may contradict current practice or policy in some jurisdictions. As such, some providers may feel discomfort about de-implementing screening, owing to concerns about missing cases of depression in this population. However, given the accuracy of the available screening instruments, providers should be aware that some cases would still be missed by screening (false negatives) and many positive screens will be false positives.^22^

The impact on equity of a recommendation against screening is unknown. Some marginalized individuals report barriers to disclosing depressive symptoms or concerns with their health care provider (e.g., being unsure how to bring up the topic of depression, concerns about stigma, aversion to antidepressant medications or psychotherapy),^23^ in which case a recommendation against screening may result in some individuals with depression not being identified.

### Rationale

This conditional recommendation is based on the very low–certainty evidence on the effect of screening on benefit outcomes and limited evidence of harms. The supporting systematic review suggests that the additional benefit of screening all patients with a questionnaire with a cut-off score compared with usual care (which should include inquiry into mood and mental health) during primary care visits is very uncertain.^19^ Although no evidence was found on the harms of screening in our systematic review, evidence from other sources described below suggests the time and focus on screening could reduce opportunities to discuss other aspects of health during a perinatal primary care encounter, as providers would be evaluating and potentially referring all patients who screen positive, in many cases unnecessarily. Screening could lead to an increase in false positives, false negatives, unnecessary referrals and diagnostic evaluation, and overdiagnosis for some patients.

A false positive can occur when the patient meets a screening cut-off score and is sent for additional psychiatric evaluation, which finds they do not actually meet the diagnostic criteria for depression. A recent individual patient data meta-analysis provides accuracy information for the EPDS, the tool used in the 1 trial we identified.^22^ Based on a prevalence of depression of 8%, screening 100 patients with the EPDS using the common cut-off score of 13 would result in 5 true positives, 3 false negatives, 5 false positives and 87 true negatives.^22^,^24^ This means that some patients who are screened will be sent for an unnecessary additional assessment.

Overdiagnosis could occur in patients with mild temporary symptoms, who might meet a screening cut-off score, leading to further evaluation and possible referral to specialty mental health services, but who would not benefit as the symptoms would subside on their own.^25^ Given the substantial challenges to accessing mental health services in Canada,^26^,^27^ the unnecessary redirection of resources from the treatment of patients with mental health disorders could be an unintended harm of screening. In a 15-minute clinical encounter, even 1–2 minutes spent reviewing the results of a formal screening instrument without proven value comprises a substantial amount of time. In the task force’s view, this could detract from the ability of the clinician to have a meaningful and empathetic discussion about the health of the patient.

As noted above, about 10% of all patients screened using a questionnaire and cut-off score would have to receive additional assessment or referrals, and thus the resource implications also extend beyond the initial clinical encounter. The task force is mindful of the resource constraints faced by Canada’s primary health care systems and as such makes recommendations against interventions when the resource implications of a particular health intervention are certain to be important and benefits have not been demonstrated.^17^

## Methods

The task force is an independent panel of clinicians and scientists that makes recommendations on primary and secondary prevention in primary care (http://www.canadiantaskforce.ca). A working group of 5 members of the task force (H.C., E.L., J.C.L., A.M., J.J.R.) developed this recommendation with scientific support from Public Health Agency of Canada (PHAC) staff.

The recommendation was informed by a systematic review that addressed the benefits and harms of screening for depression in pregnant and postpartum individuals (see analytic framework in protocol)^28^ as well as patient engagement activities that addressed specific aspects of guideline development.

The Evidence Review and Synthesis Centre at the Ottawa Hospital Research Institute conducted the systematic review according to a published protocol.^28^ Peer-reviewed search strategies were conducted in MEDLINE, Embase, PsycINFO, CINAHL and the Cochrane Library, from database inception to May 2020, with supplemental searches for grey literature. A subsequent search update to Mar. 27, 2022, was conducted in these databases before publication, which did not identify any additional studies. Studies were excluded if they recruited patients with a recent history or current diagnosis of depression, patients receiving treatment for depression or other mental disorders, and patients who were receiving services owing to mental health symptoms, as well as studies where patients were being assessed in psychiatric or mental health settings. Potential benefits of screening examined in the systematic review included symptoms or diagnosis of depression, health-related quality of life, reported or observed capacity to parent, suicidality, relationships with partners, interactions between child-bearing individual and child, infant health and development (e.g., developmental delay, birth weight), and infant responsiveness. Potential harms of screening examined were false positives, overdiagnosis or overtreatment, harms of being labelled or stigma, and harms of treatment.

The working group rated the importance of outcomes according to the Grading of Recommendations, Assessment, Development and Evaluation (GRADE) approach.^15^ Outcomes rated as critical or important by focus group participants (described below) and working group members were considered during guideline development. The working group also used the GRADE approach to determine the certainty of the evidence and strength of the recommendation (Box 1). Appendix 1 (available at www.cmaj.ca/lookup/doi/10.1503/cmaj.220290/tab-related-content) provides the evidence-to-decision framework that the task force used to develop the recommendation. The entire task force approved the recommendations.

More information about the task force’s methods is available at the task force website (https://canadiantaskforce.ca/methods/).

### Patient engagement

Patients were engaged in guideline development, recruited via advertisements on public advertisement websites (i.e., Craigslist and Kijiji), through 2 phases of focus groups conducted by the Knowledge Translation group at St. Michael’s Hospital. During phase 1 (data were collected between May 7 and June 8, 2018), 15 participants (6 pregnant and 9 postpartum, all identifying as female) assessed the importance of key outcomes in deciding whether to be screened for depression via online survey. This was followed by 3 focus groups (*n* = 13) and 2 interviews (*n* = 2) via teleconference to gather these participants’ rationale for their ratings and discuss factors that affected the importance of outcomes.^20^ In phase 2 (data were collected between May 14 and July 3, 2019), 14 different participants (4 pregnant and 10 postpartum, all identifying as female) were asked to rate the importance of outcomes when presented with synthesized evidence for benefits and harms of depression screening via online survey. This was followed by 4 focus groups (*n* = 12) and 2 interviews (*n* = 2) via teleconference to gather participants’ rationale for their ratings and discuss factors that affected the importance of outcomes.^21^

The Knowledge Translation Program at St. Michael’s Hospital (Toronto) developed knowledge translation tools to accompany this guideline, which can be found on the task force website (http://www.canadiantaskforce.ca). The tools were informed by feedback from clinicians and patients.

### External and content expert review

The protocol,^28^ systematic review^19^ and draft guideline were externally reviewed by academic peer reviewers and organizational stakeholders (see Acknowledgements). The task force also engaged clinical and content experts who helped the task force to understand important clinical issues and address technical concerns, participated in working group discussions and reviewed key supporting documents, including the final guideline. Clinical and content experts do not provide input into or vote on task force recommendations.

### Management of competing interests

The task force follows Guidelines International Network principles for disclosure of interests and management of conflicts of interest.^29^,^30^ The task force’s oversight committee for evaluating and adjudicating competing interests consists of the task force chair and vice-chair and the director of the Global Health and Guidelines Division of PHAC.^30^ Funding for the task force is provided by PHAC. The task force does not consider the views of the funding body in developing its recommendations.

All task force members are required to disclose financial and other relevant interests, and these are available on the task force website (https://canadiantaskforce.ca/about/members/). Clinical and content experts also disclose relevant interests at the outset of their participation and annually thereafter. Information on disclosures and conflicts of interest can be found in Appendix 2 (available at www.cmaj.ca/lookup/doi/10.1503/cmaj.220290/tab-related-content). Scott Klarenbach, who was a task force member but not a member of the topic working group, is the director of the Real World Evidence Unit, University of Alberta, and director and co-chair of the Real World Evidence Consortium (with University of Calgary and Institute of Health Economics). Although he receives no personal honoraria, the group relies in part on industry funding. This was not judged as a conflict when initially disclosed. However, between the initial submission of this guideline to *CMAJ* and revision after peer review, the task force oversight committee made the decision that Dr. Klarenbach should not vote on the revised guideline. He did not vote on any changes made to the guideline in response to peer review, or approve resubmission, and as such is not listed as a contributing author.

No other declared interests affected participation in the guideline development process.

## Implementation

The term “screening” in this recommendation refers to a routine process in which primary care providers administer an instrument such as a questionnaire to every pregnant or postpartum individual not already reporting symptoms of depression, and then use a cut-off score to determine a follow-up action for those at or above the cut-off score.^7^–^9^ The task force recommends against the addition of such a screening process because of the absence of evidence that it adds value beyond discussions about overall well-being, depression, anxiety and mood that are currently a part of established perinatal clinical care.

Ten provinces and territories in Canada provide guidance documents (e.g., best practice recommendations, care pathways, perinatal records) that suggest asking patients about current depression, anxiety or mood during pregnancy or the postpartum period as part of usual clinical care (Appendix 3, available at www.cmaj.ca/lookup/doi/10.1503/cmaj.220290/tab-related-content; Appendix 4, available at www.cmaj.ca/lookup/doi/10.1503/cmaj.220290/tab-related-content). Nine provinces and territories provide guidance documents that suggest primary care providers (e.g., public health nurses, family physicians, midwives) screen patients with instruments such as the EPDS during pregnancy or the postpartum period (Appendix 3; Appendix 5, available at www.cmaj.ca/lookup/doi/10.1503/cmaj.220290/tab-related-content). This guidance includes recommended cut-off scores and follow-up actions as part of screening. Among these 9 provinces and territories, 7 also provide a place to enter scores for depression screening instruments in medical record forms used during pregnancy or the postpartum period (Appendices 3 and 5). Guidance on which questionnaires to use, when to administer them and predefined cut-off scores varies across provinces and territories.

Some jurisdictions in Canada include screening as part of their standard perinatal care without fully defining it as such, whereas we use the term “screening” to indicate a comprehensive, systematic process applied in a standard way and with defined follow-up diagnostic processes for all individuals in a defined group (e.g., all patients in a clinical setting with specific characteristics). As screening practices vary across Canada (Appendices 3, 4 and 5), jurisdictions may reconsider the use of such screening in settings where it is currently implemented.

As noted above, the task force definition of screening in this context means that the recommendation against screening emphasizes the importance of good clinical practice, in which clinicians inquire about and are alert to changes in physical and mental health symptoms of their patients. Given the health implications of depression during pregnancy and the postpartum period, it is essential that providers inquire about and be attentive to mental health and well-being. If providers are uncertain about how to engage in these discussions with patients, they may consider referring to questionnaires for discussion prompts (without engaging in formal screening by using the questionnaire score for determining subsequent actions).

### Monitoring and evaluation

Clinician awareness of this recommendation against screening is a performance measure for this guideline. The task force will monitor evidence related to this guideline and will update the recommendation if new evidence becomes available that could influence its direction or strength.

## Other guidelines

Table 1 presents recommendations on screening for depression during pregnancy or the postpartum period from 6 other systematically developed provincial and national clinical practice guidelines (distinguished from the guidance documents summarized in Appendix 3).^36^ Three guidelines recommend screening of all pregnant and postpartum individuals,^10^,^31^,^35^ but they are not based on direct evidence of benefit from screening. Timing of screening, use of questionnaires and cut-off scores differ among guidelines.

**Table 1:** Recommendations on screening for depression in pregnant and postpartum populations

Organization	Recommendation
Canadian Task Force on Preventive Health Care (current guideline, 2022)	The task force recommends against instrument-based depression screening using a questionnaire with cut-off score to distinguish “screen positive” and “screen negative” administered to all individuals during pregnancy and the postpartum period (up to 1 year after childbirth) (conditional recommendation, very low–certainty evidence).
Canadian Task Force on Preventive Health Care (2013)^12^	For adults in subgroups of the population who may be at increased risk of depression,* we recommend not routinely screening for depression (weak recommendation; very-low-quality evidence).
Registered Nurses’ Association of Ontario (Canada)^31^	Routinely screen for risk of perinatal depression, using a valid tool, as part of prenatal and postpartum care.
US Preventive Services Task Force (United States)^10^	Screening for depression is recommended in the general adult population, including pregnant and postpartum women. Screening should be implemented with adequate systems in place to ensure accurate diagnosis, effective treatment, and appropriate follow-up.
UK National Screening Committee (United Kingdom)^32^	A systematic antenatal and postnatal population screening program for mental health problems is not recommended.
National Institute for Health and Care Excellence (England)^9^	At a woman’s first contact with primary care or her booking visit, and during the early postnatal period, consider asking the following depression identification questions as part of a general discussion about a woman’s mental health and well-being: During the past month, have you often been bothered by feeling down, depressed or hopeless? During the past month, have you often been bothered by having little interest or pleasure in doing things? If a woman responds positively to either of the depression identification questions, is at risk of developing a mental health problem, or there is clinical concern, consider using the EPDS or the Patient Health Questionnaire as part of a full assessment or referring the woman to her general practitioner or, if a severe mental health problem is suspected, to a mental health professional.
Scottish Intercollegiate Guidelines Network (Scotland)^33^	Enquiry about depressive symptoms should be made, at minimum, on booking in and postnatally at 4–6 weeks and 3–4 months. The EPDS or the Whooley Questions^34^ may be used in the antenatal and postnatal period as an aid to clinical monitoring and to facilitate discussion of emotional issues.
Centre of Perinatal Excellence (Australia)^35^	Use the EPDS to screen women for a possible depressive disorder in the perinatal period. Complete the first antenatal screening as early as practical in pregnancy and repeat screening at least once later in pregnancy. Complete the first postnatal screening 6–12 weeks after birth and repeat screening at least once in the first postnatal year. Arrange further assessment of perinatal woman with an EPDS score of 13 or more.

Note: EPDS = Edinburgh Postnatal Depression Scale.

* Subgroups of the population who may be at increased risk of depression include people with a family history of depression, traumatic experiences as a child, recent traumatic life events, chronic health problems, substance misuse or Indigenous origin.

The UK National Screening Committee recommends against a systematic antenatal and postnatal population screening program for mental health problems.^32^ The 2 remaining guidelines recommend considering administering questions to identify depression as part of a general discussion of mental well-being^9^ and enquiry into depressive symptoms during pregnancy and postpartum.^33^

## Gaps in knowledge

Only 1 RCT has assessed the benefits and harms of questionnaire-based screening for depression versus no screening during the postpartum period. None have assessed such screening during pregnancy. Trials that compare screening to usual clinical care, where participants identified as depressed in either arm receive the same level of care, are needed in order to isolate the effectiveness of screening as an intervention. Outcomes should include both maternal- and infant-related benefits and harms. As experiences of pregnancy and the postpartum period can vary based on factors such as culture, ethnicity, socioeconomic status, geographic region and other social determinants of health,^6^ studies that reflect and provide evidence for the diversity of the Canadian population would also be helpful.

## Limitations

Although we sought patient values and preferences through our patient engagement activities, the results of this work should be interpreted and generalized with caution given the small sample sizes. We did not examine the peer-reviewed evidence base for patient values and preferences.

Assessment of current practices in Canada (Appendices 3, 4 and 5) is based on publicly available documents, and actual current practices may vary.

## Conclusion

Overall, the effect of screening for depression during pregnancy and the postpartum period was very uncertain on all critical outcomes examined. In the judgment of the task force, there are also important resource implications to screening using an unproven screening instrument. Therefore, the task force conditionally recommends against screening during pregnancy and the postpartum period for depression, using questionnaires with cut-off scores, among individuals without a personal history of depression, or who are not already being assessed or treated for other mental disorders.

## Supplementary Material

Appendix 1: Screening for Depresion in Pregnancy/Postpartum Evidence-to-Decision Framework

Appendix 2. Summary of disclosures of interest for this guideline

Appendix 3

Appendix 4. Asesing Depresion During the Pregnancy and Postpartum Period by Province/Territory

Appendix 5. Use of EPDS During Pregnancy and/or Postnatal Acros Provinces and Territories
