# Supplementary material for: Recommendation on instrument-based screening for depression during pregnancy and the postpartum period
Source: CMAJ. 2022 Jul 25;194(28):E981–9. doi: 10.1503/cmaj.220290 (PMC9328462; doi:10.1503/cmaj.220290)
Supplement: Appendix 1: Screening for Depresion in Pregnancy/Postpartum Evidence-to-Decision Framework [file 220290-guide-1-at.pdf]

Appendix 1: Screening for Depression in Pregnancy/Postpartum Evidence-to-Decision Framework

Should people be additionally screened for depression during pregnancy and the postpartum period (one year following childbirth)?

POPULATION: Individuals of any age during pregnancy and up to one year postpartum

INTERVENTION: Interventions that use a single question, small sets of questions, or a screening questionnaire (validated or non-validated) with a pre-defined cut-off score to identify patients who may have depression, but who have not reported their symptoms to healthcare providers or who have otherwise not been identified as possibly depressed by healthcare providers. Exceeding a cut-off score must be followed by a specified action such as more thorough assessment or referral.

COMPARISON: No depression screening. Usual care during pregnancy and the postpartum period typically includes informally asking pregnant and postpartum individuals about their personal and family history of mental illness (e.g., anxiety, depression) and current mood-related symptoms.

MAIN OUTCOMES:

Critical:

- Symptoms of depression (using continuous or dichotomous measures) or a diagnosis of major depressive disorder (MDD) using a validated diagnostic interview
- Health-related quality of life (using validated tools)
- Reported or observed capacity to parent (e.g., attachment, responsiveness to infant, positive regard of infant/fetus)

Important:

- Suicidality (suicidal ideation, attempt, or completion)
- False positive screens (positive screens in absence of depressive disorder), overdiagnosis, or overtreatment
- Harms of being labelled/stigma
- Harms of treatment
- Relationship with partner and other supports
- Mother-child interactions including mutual touching, smiling, vocalizations
- Infant health and development (e.g., developmental delay, failure to thrive, cognitive, emotional, motor and neural functioning and development
- Infant responsiveness

SETTING: Primary care clinical settings

PERSPECTIVE: Population

BACKGROUND

Depression in pregnancy or in the postpartum period (within one year after childbirth) can be a serious illness that can result in burdensome symptoms (e.g., sad mood most of the day, loss of interest in almost all activities and sleep disturbances). It also can be associated with negative health outcomes for both mother (e.g., reduced quality of life, suicidal thoughts or actions) and child (e.g., impaired physical, mental and emotional development).
Having depression while pregnant, or as a new mother, can negatively affect other areas of life including close relationships, parenting abilities, and mother-child interactions.

Unlike usual communication and discussion during a patient visit, screening for depression involves <u>formally asking all patients a defined set of questions or administering a questionnaire with a pre-defined cut-off score</u> to identify patients who may have depression. Patients with positive screening scores must be followed up by a health care provider to conduct a more detailed mental health assessment or make an appropriate referral.
Treatment for depression during pregnancy and the postpartum period can include supportive interventions, psychotherapy, lifestyle changes, and antidepressant medications.

Appendix 1, as supplied by the authors. Appendix to: Lang E, Colquhoun H, LeBlanc JC, et al; Canadian Task Force on Preventive Health Care Recommendation on instrument-based screening for depression during pregnancy and the postpartum period. *CMAJ* 2022. doi: 10.1503/cmaj.220290. Copyright © 2022 The Author(s) or their employer(s). To receive this resource in an accessible format, please contact us at cmajgroup@cmaj.ca.

Assessment

	JUDGEMENT	RESEARCH EVIDENCE	ADDITIONAL CONSIDERATIONS
PROBLEM	**Is the problem a priority?**<br>○ No<br>○ Probably no<br>○ Probably yes<br>**X Yes**<br><br>○ Varies<br>○ Don't know	A systematic review from 2005 estimated that the point prevalence of major depression at different periods of pregnancy and postpartum ranges from 1% to 6% at different time points, based on 2-6 studies at any given time point (111 to 2104 participants)(1). A 2008 national survey from the USA of over 14,000 participants aged 18 to 50 years reported that the 12-month period prevalence of depression was 8% among pregnant individuals and 9% in postpartum individuals, compared to 8% among non-pregnant individuals (2). Depression during the postpartum period can have far-reaching impacts on the childbearing parent and their infant, affecting parent-infant interactions and relationships with partners (4). If left untreated, depression can have significant consequences such as reduced breastfeeding, poor maternal-infant bonding, and developmental delays for the infant (4,5).	

DESIRABLE EFFECTS

JUDGEMENT

How substantial are the desirable anticipated effects?
○ Little to none
○ Small
○ Moderate
○ Large

○ Varies
X Don't know

RESEARCH EVIDENCE

The evidence review did not identify any studies of screening for depression during pregnancy.

The evidence review found one study of screening for depression among postpartum individuals (described as “mothers” or “women” in the study). Evidence from 1 RCT (n=462) indicated that the effects of screening on <u>the number of patients identified as depressed, depression scores, and minor psychiatric conditions</u> at 6-months follow-up (4-months post screening) were very uncertain (5).

The low-certainty evidence for mean infant body weight (kg) at six months suggests that screening for depression may lead to little to no difference compared to no screening (mean weight difference 0.05 kg higher, 95% CI 0.1 kg lower to 0.2 kg higher; SMD 0.06 higher, 95% CI 0.12 lower to 0.24 higher) (5).

Results from this study show that the effects of screening compared to usual care for the following outcomes was very low-certainty:
- relationship with partner and other supports (e.g., marital satisfaction scores);
- reported/observed capacity to parent (e.g., parenting stress scores, parental distress scores);
- childbearing parent-child interactions (e.g., parent-child dysfunctional interaction scores);
- infant outcomes (e.g., number of child hospitalizations).The evidence review did not identify any studies that reported on suicidality.

Table 1: Benefits of Screening

Certainty assessment							№ of patients		Effect			Certainty	Importance
№ of studies	Study design	Risk of bias	Inconsistency	Indirectness	Imprecision	Other considerations	Screening	No screening	Relative (95% CI)	Absolute (95% CI) [MD]	Absolute (95% CI) [SMD]		
Number identified as depressed (follow up: 6 months; assessed with: EPDS score ≥ 10)													
1	randomized trials	very serious [a,b]	not serious	not serious	serious [c]	none	30/231 (13%)	51/231 (22%)	-	**91 fewer per 1,000** (from 24 fewer to 135 fewer)	-	⊕◯◯◯ VERY LOW	CRITICAL
EPDS score (follow up: 6 months; assessed with: EPDS; Scale from: 0 to 30 (worse))													
1	randomized trials	very serious [a,b]	not serious	not serious	serious [c]	none	231	231	-	MD **1.36 lower** (0.63 lower to 2.09 lower)	SMD **0.34 lower** (0.15 lower to 0.52 lower)	⊕◯◯◯ VERY LOW	CRITICAL
GHQ score (follow up: 6 months; assessed with: GHQ-12; Scale from: 0 to 12 (more severe))													

1	randomized trials	very serious [a,b]	not serious	not serious	serious [c]	none	231	231	-	MD **0.33 lower** (0.7 lower to 0.04 higher)	SMD **0.16 lower** (0.35 lower to 0.02 higher)	⨁◯◯◯ VERY LOW	CRITICAL
Parenting Stress Index (PSI) total score (follow up: 6 months; assessed with: PSI-SF; Scale from: 36 to 180 (more stress))													
1	randomized trials	very serious [a,b]	not serious	not serious	serious [c]	none	231	231	-	MD **2.78 lower** (5.74 lower to 0.18 higher)	SMD **0.17 lower** (0.35 lower to 0.01 higher)	⨁◯◯◯ VERY LOW	IMPORTANT
PSI-Parental Distress (PD) subscale score (follow up: 6 months; assessed with: PSI-SF PD subscale; Scale from: 12 to 60 (more stress))													
1	randomized trials	very serious [a,b]	not serious	not serious	serious [c]	none	231	231	-	MD **1.21 lower** (2.48 lower to 0.06 higher)	SMD **0.17 lower** (0.36 lower to 0.01 higher)	⨁◯◯◯ VERY LOW	IMPORTANT
PSI-Parent-Child Dysfunctional Interaction (PCDI) subscale score (follow up: 6 months; assessed with: PSI-SF PCDI subscale; Scale from: 12 to 60 (more stress))													
1	randomized trials	very serious [a,b]	not serious	not serious	serious [c]	none	231	231	-	MD **1.08 lower** (2.17 lower to 0.01 higher)	SMD **0.18 lower** (0.36 lower to 0.00)	⨁◯◯◯ VERY LOW	IMPORTANT
PSI-Difficult Child (DC) subscale score (follow up: 6 months; assessed with: PSI-SF DC subscale; Scale from: 12 to 60 (more stress))													
1	randomized trials	very serious [a,b]	not serious	not serious	serious [c]	none	231	231	-	MD **0.49 lower** (1.64 lower to 0.66 higher)	SMD **0.08 lower** (0.26 lower to 0.10 higher)	⨁◯◯◯ VERY LOW	IMPORTANT
Marital satisfaction score (follow up: 6 months; assessed with: Chinese Kansas Marital Satisfaction Scale (CKMSS); Scale from: 3 to 21 (more satisfied))													
1	randomized trials	very serious [a,b]	not serious	not serious	serious [c]	none	231	231	-	MD **0.47 higher** (0.09 lower to 1.03 higher)	SMD **0.15 higher** (0.03 lower to 0.34 higher)	⨁◯◯◯ VERY LOW	IMPORTANT
Child body weight (in kg) (follow up: 6 months; assessed with: Maternal and Child Health Centres records)													
1	randomized trials	serious [b]	not serious	not serious	serious [c]	none	231	231	-	MD **0.05 higher** (0.1 lower to 0.2 higher)	SMD **0.06 higher** (0.12 lower to 0.24 higher)	⨁⨁◯◯ LOW	IMPORTANT
Child number of hospitalization (follow up: 6 months; assessed with: Mothers or caregivers)													
1	randomized trials	very serious [b,e]	not serious	not serious	serious [c]	none	231	231	-	MD **0.04 higher** (0.09 lower to 0.17 higher)	SMD **0.06 higher** (0.13 lower to 0.24 higher)	⨁◯◯◯ VERY LOW	IMPORTANT

Explanations (Tables 1 and 2)

a. Questionnaire was completed by participants.

b. Selective reporting identified. One of the primary outcomes listed in clinicaltrials.gov was listed as a secondary outcome in the publication. The primary outcome reported as such was statistically significant, and the other was not statistically significant.

c. Due to uncertainty and lack of empirical evidence to support, we have elected not to calculate an optimal information size Further, GRADE suggests a rule of thumb of a minimum of 400 events for dichotomous

 Copyright © 2022 The Author(s) or their employer(s). To receive this resource in an accessible format, please contact us at cmajgroup@cmaj.ca.

outcomes and 400 participants for continuous outcomes. As there were 462 participants in this study and without a known threshold for continuous outcomes, a judgement of “serious concern” was applied.

d. No information on how this information was collected, so many domains were unclear.

e. Reported by participants/caregivers.

UNDESIRABLE EFFECTS

How substantial are the undesirable anticipated effects?
○ Large
○ Moderate
○ Small
○ Little to none

○ Varies
X Don't know

No trials that met inclusion criteria assessed the harms of screening for depression during pregnancy or the postpartum period (e.g., false positives, overdiagnosis, overtreatment, or labeling/stigma). In one RCT, no adverse events were reported in either group of screened and not screened participants (5). However, it was unclear how adverse events were defined, collected, and reported in the study so the effect could not be estimated and evidence was considered very low-certainty.

The evidence review did not assess harms for infants related to screening their childbearing parent for depression during pregnancy or the postpartum period.

Table 2: Harms of Screening

Certainty assessment							№ of patients		Effect			Certainty	Importance
№ of studies	Study design	Risk of bias	Inconsistency	Indirectness	Imprecision	Other considerations	Screening	No screening	Relative (95% CI)	Absolute (95% CI) [MD]	Absolute (95% CI) [SMD]		
Adverse events (follow up: 6 months postpartum; assessed with: not reported)													
1	randomized trials	very serious [b,d]	very serious [b,d]	not serious	not serious	serious [c]	231	231	-	Not estimable	Not estimable	⊕◯◯◯ VERY LOW	IMPORTANT

Explanations (Tables 1 and 2)
a. Questionnaire was completed by participants.
b. Selective reporting identified. One of the primary outcomes listed in clinicaltrials.gov was listed as a secondary outcome in the publication. The primary outcome reported as such was statistically significant, and the other was not statistically significant.
c. Due to uncertainty and lack of empirical evidence to support, we have elected not to calculate an OIS. Further, GRADE suggests a rule of thumb of a minimum of 400 events for dichotomous outcomes and 400 participants for continuous outcomes. As there were 462 participants in this study and without a known threshold, a judgement of serious concern was applied.
d. No information on how this information was collected, so many domains were unclear.
e. Reported by participants/caregivers.

CERTAINTY OF EVIDENCE

What is the overall certainty of the evidence of effects?
X Very low
○ Low
○ Moderate
○ High

○ No included studies

There is very low certainty that the evidence of effects represents the true effects of screening people for depression during pregnancy or up to one year postpartum. We are very uncertain about the absolute effects of screening on the critical outcomes of interest. This means that the true effects of screening are likely substantially different from the study data.

The very serious concerns with Risk of Bias with the Leung (2010) study include: Blinding of outcome assessors (self-report questionnaires) and selective outcome reporting (one of the primary outcomes listed in the protocol was reported as a secondary outcome in the study publication) (5).

There were also serious concerns with imprecision due to small sample size (n=462) (5).

VALUES

Is there important uncertainty about or variability in how much people value the main outcomes?
○ Important uncertainty or variability
○ Possibly important uncertainty or variability
X Probably no

A systematic review on patients’ values and preferences was not conducted.

Two phases of knowledge translation activities were conducted among pregnant and postpartum individuals.

KT Phase 1 results (6):

• A total of 15 participants (6 currently pregnant, 9 postpartum, all identifying as female gender) were recruited to participate in the online surveys and focus group discussions via teleconference to rate the importance of key outcomes. Most participants were from Ontario (n=9), with others from British Columbia (n=5) and Alberta (n=1). A total of 3 participants reported previous or current diagnoses of or treatment for depression.
• Suicidality and 3 infant-related outcomes (infant health and development; mother-child interactions; capacity to parent) were rated the most critical to consider when deciding whether or not to get screened for depression.
• Harms of screening (false positives, overdiagnosis, overtreatment, and labelling) were rated the lowest (but still

Appendix 1, as supplied by the authors. Appendix to: Lang E, Colquhoun H, LeBlanc JC, et al; Canadian Task Force on Preventive Health Care Recommendation on instrument-based screening for depression during pregnancy and the postpartum period. *CMAJ* 2022. doi: 10.1503/cmaj.220290. Copyright © 2022 The Author(s) or their employer(s). To receive this resource in an accessible format, please contact us at cmajgroup@cmaj.ca.

	**important uncertainty or variability**<br>○ No important uncertainty or variability	'important').<br>• There was a very strong preference to be screened for depression among participants (median score to the question: "…how much would you want to be screened during pregnancy or the postpartum period" was '9 = Very Much').<br>• Many believed that screening at several times during pregnancy/postpartum period, and by several health care providers (e.g., midwives, doulas, nurse practitioners, pediatricians), would be beneficial.<br><br>KT Phase 2 results (7):<br><br>• A total of 14 participants (4 currently pregnant, 10 postpartum, all identifying as female gender) were recruited to participate in the online surveys and focus group discussions via teleconference to rate the importance of key outcomes. Participants were from Ontario (n=7), British Columbia (n=4), Quebec (n=1), Prince Edward Island (n=1), and New Brunswick (n=1). A total of 5 participants reported a previous diagnosis or treatment of depression by a health professional and 2 participants reported currently receiving treatment for depression.<br>• In the final survey, participants rated almost all screening benefits as 'critical' to consider during decision making, whereas they rated all screening harms slightly lower, but still 'important'.<br>• Participants reported a preference for screening. When asked "Considering the risk of many of the harms and benefits of screening for depression during the pregnancy and postpartum period are not well known, how much would you want to be screened", participants median score was 7.5, where 5='Neutral' and 9='Very Much'.<br>• "[W]hile participants rated their preference to be screened fairly highly in the survey, focus group discussions indicated that participants felt most strongly about having a discussion with a healthcare provider about their mental health and wellbeing, rather than a formal screening process. They felt a discussion about depression with a primary health care provider during the pregnancy and [postpartum] period is critical[.]"<br>• "[Participants] identified types of information that would be helpful as part of a screening discussion, including: differentiating between depression and the "baby blues", providing information on the prevalence of depression in the pregnancy and [postpartum] period, describing symptoms to be aware of, and describing potential next steps following a diagnosis (e.g., duration and availability of treatment)."<br>• "Participants expressed concerns that without screening, they may not be capable of identifying symptoms of depression, or may not take initiative to seek input from a primary care provider, especially considering all of the stresses and changes one goes through as a new parent."	
BALANCE OF EFFECTS	**Does the balance between desirable and undesirable effects favor the intervention or the comparison?**<br>○ Favors the comparison<br>○ Probably favors the comparison<br>**X Does not favor either the intervention or the comparison**<br>○ Probably favors the intervention<br>○ Favors the intervention<br><br>○ Varies<br>○ Don't know	The evidence was very uncertain from this single RCT regarding whether screening for depression may be beneficial for reducing the number of postpartum individuals classified as having depression 4 months after screening or for reducing their EPDS score (5).<br><br>The same study reported very low-certainty evidence on differences between those screened and not screened for the outcomes: mental health symptoms (as measured by the General Health Questionnaire), their relationship with partner and other supports, reported/observed capacity to parent, childbearing parent-child interactions, or infant outcomes (number of hospitalizations) (5). Low-certainty evidence suggested little to no effect of screening on infant body weight (5).<br><br>No evidence was found on the undesirable effects of screening such as suicidality, false positives, overdiagnosis, overtreatment, or labeling/stigma. No adverse events in either the screened and not screened group were recorded in the one RCT identified in the systematic review (5).	
RESOURCES REQUIRED	**How large are the resource requirements (costs)?**<br>○ Large costs<br>**X Moderate costs**<br>○ Negligible costs / savings<br>○ Moderate savings<br>○ Large savings<br><br>○ Varies<br>○ Don't know	If we choose a Recommendation FOR Screening:<br>There would be costs associated with a recommendation for routine screening of patients for depression during pregnancy and the postpartum period. These costs could include items such as: clinician and patient time, printing and technological costs of administering the screening instrument, and further costs related to increased demand for psychiatry/psychology resources, and treatment (e.g., counselling and/or medications). However, the total costs of a depression screening program in Canada are not known.<br><br>If we choose a Recommendation AGAINST Screening:<br>The resource requirements for following a recommendation against screening are unknown. It is possible that resource savings (e.g., primary care provider time, unnecessary follow-up and treatment costs) could occur in primary care settings that had previously administered formal screening instruments to all patients during pregnancy and the postpartum period. However, implications may vary depending on current practice and are not known with certainty.	

 Copyright © 2022 The Author(s) or their employer(s). To receive this resource in an accessible format, please contact us at cmajgroup@cmaj.ca.

CERTAINTY OF EVIDENCE OF REQUIRED RESOURCES	**What is the certainty of the evidence of resource requirements (costs)?**<br>○ Very low<br>○ Low<br>○ Moderate<br>○ High<br><br>**X No included studies**	A cost-effectiveness systematic review was not completed. However, given that universal screening is not current practice in all jurisdictions, implementation of screening will certainly include some costs (the exact costs are unknown).	
COST EFFECTIVENESS	**Does the cost-effectiveness of the intervention favor the intervention or the comparison?**<br>○ Favors the comparison<br>○ Probably favors the comparison<br>○ Does not favor either the intervention or the comparison<br>○ Probably favors the intervention<br>○ Favors the intervention<br><br>○ Varies<br>**X No included studies**	A cost-effectiveness systematic review was not completed.	
EQUITY	**What would be the impact on health equity?**<br>○ Reduced<br>○ Probably reduced<br>○ Probably no impact<br>○ Probably increased<br>○ Increased<br><br>○ Varies<br>**X Don't know**	A recommendation FOR screening: there could be an impact on equity if screening is implemented and resources are redirected away from treatment of patients with known mental health disorders who often do not receive adequate treatment and are re-allocated to universal screening.<br><br>It is not known what impact a recommendation AGAINST screening would have on equity. Some marginalized women report barriers to disclose depressive symptoms/concerns with their health care provider, in which case a recommendation against formal screening may result in some women with depression not being identified.	
ACCEPTABILITY	**Is the intervention acceptable to key stakeholders?**<br>○ No<br>○ Probably no<br>○ Probably yes<br>○ Yes<br><br>**X Varies**<br>○ Don't know	Is a recommendation FOR screening acceptable to key stakeholders?<br>Many clinicians routinely and informally ask pregnant and postpartum patients how they are feeling (e.g., during well-baby visits). A recommendation for screening would mechanize this process for all patients and it would increase referrals/wait lists for those that screened positive and require further assessment, many of whom would not end up meeting the clinical diagnosis of depression. A recommendation for screening may not be acceptable to stakeholders if this leads to an increase in referrals/wait lists that would consume specialist resources that could be used for patients with more severe mental health care needs.<br><br>Is a recommendation AGAINST screening acceptable to key stakeholders?<br>The recommendation against systematically using formal, instrument-based screening using a questionnaire of all pregnant and postpartum patients would be acceptable to most patients as long as providers continue to inquire about mental health and wellbeing as part of usual care. In patient engagement activities supporting this guideline, participants reported a preference for screening but felt most strongly about having a discussion regarding mental health and wellbeing with	We have not assessed whether the screening questionnaires themselves are acceptable to patients.

 Copyright © 2022 The Author(s) or their employer(s). To receive this resource in an accessible format, please contact us at cmajgroup@cmaj.ca.

		their healthcare provider. In the judgement of the task force, discussions regarding mental health and wellbeing can occur within the context of usual care and are consistent with a recommendation against screening.<br><br>The task force believes this recommendation would be acceptable to some stakeholders, such as primary care providers and policymakers, as it highlights the lack of evidence to support screening but affirms the clinical practice of inquiring into depression, anxiety, and mood. However, a recommendation against screening may contradict current practice or policy in some jurisdictions. As such, some providers may feel discomfort about de-implementing screening due to concerns about 'missing' cases of this important health issue. However, based on the evidence examined, carrying out formal screening is not an effective solution to this concern.<br><br>Note that a recommendation against screening is not a recommendation against the role of clinicians in alerting patients about mood changes that can occur during pregnancy and the postpartum period.	
FEASIBILITY	**Is the intervention feasible to implement?**<br>o No<br>o Probably no<br>o Probably yes<br>**X Yes**<br><br>o Varies<br>o Don't know	Recommendation FOR or AGAINST screening:<br><br>A recommendation against screening is considered feasible by the task force. We do not know the degree to which this would represent a change in practice for clinicians. While most provinces/territories have guidance that recommend using a screening instrument, we do not know how many clinicians are following this. Nevertheless, even with a recommendation against routine screening, clinicians should still remain alert to signs or symptoms of depression in their patients as part of their usual vigilance and should alert all patients that mood can change during pregnancy and the postpartum period even though these changes don't necessarily mean the patient has a depressive disorder	

Summary of judgements

	JUDGEMENT							IMPLICATIONS
**PROBLEM**	No	Probably no	Probably yes	Yes		Varies	Don't know	
**DESIRABLE EFFECTS**	Little to none	Small	Moderate	Large		Varies	Don't know	
**UNDESIRABLE EFFECTS**	Large	Moderate	Small	Little to none		Varies	Don't know	
**CERTAINTY OF EVIDENCE**	Very low	Low	Moderate	High			No included studies	
**VALUES**	Important uncertainty or variability	Possibly important uncertainty or variability	Probably no important uncertainty or variability	No important uncertainty or variability				
**BALANCE OF EFFECTS**	Favors the comparison	Probably favors the comparison	Does not favor either the intervention or the comparison	Probably favors the intervention	Favors the intervention	Varies	Don't know	
**RESOURCES REQUIRED**	Large costs	Moderate costs	Negligible costs and savings	Moderate savings	Large savings	Varies	Don't know	
**CERTAINTY OF EVIDENCE OF REQUIRED RESOURCES**	Very low	Low	Moderate	High			No included studies	
**COST EFFECTIVENESS**	Favors the comparison	Probably favors the comparison	Does not favor either the intervention or the comparison	Probably favors the intervention	Favors the intervention	Varies	No included studies	

 Copyright © 2022 The Author(s) or their employer(s). To receive this resource in an accessible format, please contact us at cmajgroup@cmaj.ca.

	JUDGEMENT							IMPLICATIONS
**EQUITY**	Reduced	Probably reduced	Probably no impact	Probably increased	Increased	Varies	Don't know	
**ACCEPTABILITY**	No	Probably no	Probably yes	Yes		Varies	Don't know	
**FEASIBILITY**	No	Probably no	Probably yes	Yes		Varies	Don't know	

Conclusions

Should people be screened for depression during pregnancy and the postpartum period?

**TYPE OF RECOMMENDATION**	Strong recommendation against the intervention	**X Conditional recommendation against the intervention**	Conditional recommendation for either the intervention or the comparison	Conditional recommendation for the intervention	Strong recommendation for the intervention
**RECOMMENDATION**	The task force recommends against instrument-based depression screening using a questionnaire with cut-off score to distinguish "screen positive" and "screen negative" administered to all individuals during pregnancy and the postpartum period (up to 1 year after childbirth) (conditional recommendation, very low-certainty evidence).<br><br>This recommendation assumes that, as part of usual care during pregnancy and the postpartum period, care providers will inquire about and be attentive to mental health and wellbeing.				
**JUSTIFICATION**	The supporting systematic review suggests that the additional benefit of screening all patients with a questionnaire with a cut-off score compared to usual care (which should include inquiry into mood and mental health) during primary care visits is very uncertain. Although no evidence was found on the harms of screening in our systematic review, evidence from other sources described below suggest the time and focus on screening could reduce opportunities to discuss other aspects of health during a perinatal primary care encounter as providers would be evaluating and potentially referring all patients who screen positive, in many cases unnecessarily. Screening could lead to an increase in false positives, false negatives, unnecessary referrals and diagnostic evaluation, and overdiagnosis for some patients.<br><br>A false positive can occur when the patient meets a screening cut-off score and is sent for additional psychiatric evaluation, which finds they do not actually meet the diagnostic criteria for depression. A recent individual patient data meta-analysis provides accuracy information for the EPDS, the tool used in the one trial we identified (8). Based on a prevalence of 8%, screening 100 patients with the EPDS using the common cut-off score of 13 would result in 5 true positives, 3 false negatives, 5 false positives, and 87 true negatives (8,9). This means that some patients who are screened will be sent for an unnecessary additional assessment.<br><br>Overdiagnosis could occur in patients with mild temporary symptoms, who might meet a screening cut-off score, leading to further evaluation and possible referral to specialty mental health services, but who would not benefit as the symptoms would subside on their own. Given the significant challenges to accessing mental health services in Canada, the unnecessary redirection of resources from the treatment of patients with mental health disorders could be an unintended harm of screening. Spending even one to two1-2 minutes per clinical encounter reviewing the results of a formal screening instrument with no proven value could consume a significant amount of time during a 15-minute encounter. In the task force's Force's view, this could detract from the ability of the clinician to have a meaningful and empathetic discussion about the health of the patient. As noted above, about 10% of all patients screened using a questionnaire and cut-off score would have to receive additional assessment or referrals, and thus the resource implications also extend beyond the initial clinical encounter. The task force is mindful of the resource constraints faced by our primary health care system and as such makes recommendations against interventions when the resource implication of a particular health intervention are certain to be important and benefits have not been demonstrated (10).				
**SUBGROUP CONSIDERATIONS**	This recommendation applies to all patients, including patients who have characteristics that may suggest elevated risk of depression (e.g., trauma in early life, family history of depression) (11).				

 Copyright © 2022 The Author(s) or their employer(s). To receive this resource in an accessible format, please contact us at cmajgroup@cmaj.ca.

	This recommendation does not apply to pregnant and postpartum individuals with a personal history or current diagnosis of depression or another mental health disorder, those currently receiving assessment or treatment for mental disorders, those receiving care in psychiatric or other mental health settings, or those who are seeking services due to symptoms of depression.
**IMPLEMENTATION CONSIDERATIONS**	The term 'screening' in this recommendation refers to a routine process in which primary care providers administer an instrument such as a questionnaire to every pregnant or postpartum individual not already reporting symptoms of depression and then use a cut-off score to determine a follow-up action for those at or above the cut-off score. The task force recommends against the addition of such a screening process because of the absence of evidence that it adds value beyond discussions about overall well-being, depression, anxiety, and mood that are currently a part of established perinatal clinical care.<br><br>Ten provinces and territories in Canada provide guidance documents (e.g., best practice recommendations, care pathways, perinatal records) that suggest asking patients about current depression, anxiety, or mood during pregnancy or the postpartum period as part of usual clinical care (Appendix 3,4). Nine provinces and territories provide guidance documents that suggest primary care providers (e.g., public health nurses, family physicians, midwives) screen patients with instruments such as the Edinburgh Postnatal Depression Scale (EPDS) during pregnancy or the postpartum period (Appendix 3,5). This guidance includes recommended cut-off scores and follow-up actions as part of screening. Among these nine provinces and territories, seven also provide a place to enter scores for depression screening instruments in medical record forms used during pregnancy or the postpartum period (Appendix 3,5). Guidance on which questionnaires to use, when to administer them, and pre-defined cut-off scores varies across provinces and territories.<br><br>Some jurisdictions included screening as part of their standard perinatal care without fully defining it as such, whereas we use the term 'screening' to indicate a comprehensive, systematic process applied in a standard way and with defined follow up diagnostic processes for all individuals in a defined group (e.g., all patients in a clinical setting with specific characteristics). As screening practices vary across Canada (Appendices 3,4,5), jurisdictions may reconsider the use of such screening in settings where it is currently implemented.<br><br>As noted above, the task force definition of screening in this context means that the recommendation against screening emphasizes the importance of good clinical practice where clinicians should inquire and be alert to changes in physical and mental health symptoms of their patients. Given the health implications of depression during pregnancy and the post-partum period, it is essential that providers inquire about and be attentive to mental health and wellbeing. If providers are uncertain about how to engage in these discussions with patients, they may consider referring to questionnaires for discussion prompts (without engaging in formal screening by using the questionnaire score for determining subsequent actions).
**MONITORING AND EVALUATION**	Clinician awareness of this recommendation against screening is a performance measure for this guideline. The task force will monitor evidence related to this guideline and will update the recommendation if new evidence becomes available that could influence its direction or strength.
**RESEARCH PRIORITIES**	There is only 1 randomized controlled trial assessing the benefits and harms of screening for depression versus no screening during the postpartum period. There were none during pregnancy. Trials that compare screening to usual clinical care, where those identified as depressed in either arm receive the same level of care, are needed in order to isolate the effectiveness of screening as an intervention. Outcomes should include both maternal and infant-related benefits and harms. As experiences of pregnancy and the postpartum period can vary based on factors such as culture, ethnicity, socioeconomic status, geographic region, and other social determinants of health (12), studies that reflect and provide evidence for the diversity of the Canadian population would also be helpful.

References

(1) Gavin NI, Gaynes BN, Lohr KN, Meltzer-Brody S, Gartlehner G, Swinson T. Perinatal depression: a systematic review of prevalence and incidence. Rockville (MD): Agency for Healthcare Research and Quality; 2005.

(2) Vesga-López O, Blanco C, Keyes K, Olfson M, Grant BF, Hasin DS. Psychiatric disorders in pregnant and postpartum women in the United States. Arch Gen Psychiatry 2008;65(7):805-15.

(3) Slomian J, Honvo G, Emonts P, Reginster J, Bruyère O. Consequences of maternal postpartum depression: A systematic review of maternal and infant outcomes. Womens Health 2019;15:1-15.

(4) Stein A, Pearson RM, Goodman SH, Rapa E, Rahman A, McCallum M, et al. Effects of perinatal mental disorders on the fetus and child. Lancet 2014;384(9956):1800-19.

 Copyright © 2022 The Author(s) or their employer(s). To receive this resource in an accessible format, please contact us at cmajgroup@cmaj.ca.

(5) Leung SSL, Leung C, Lam TH, Hung SF, Chan R, Yeung T, et al. Outcome of a postnatal depression screening programme using the Edinburgh Postnatal Depression Scale: a randomized controlled trial. J Public Health 2011;33(2):292-301.

(6) Buckland D, Scoleri R, Stein S, Einarson K, Straus S, Lang E. Patient preferences for depression screening during pregnancy and the postpartum period: Data summary. Toronto, ON; Knowledge Translation Program Li Ka Shing Knowledge Institute St. Michael's Hospital. 2018.

(7) Burnett L, Silveira K, Narcisse-Merveille C, Stein S, Einarson K, Rashid S, Straus S, Lang E, LeBlanc JC. Patient preferences for depression screening during pregnancy and the postpartum period: Phase 2 Data summary. Toronto, ON; Knowledge Translation Program Li Ka Shing Knowledge Institute St. Michael's Hospital. 2019.

(8) Levis B, Negeri Z, Sun Y, Benedetti A, Thombs B D. Accuracy of the Edinburgh Postnatal Depression Scale (EPDS) for screening to detect major depression among pregnant and postpartum women: systematic review and meta-analysis of individual participant data. BMJ 2020; 371 :m4022.

(9) Using the EPDS to Screen for Depression in Pregnancy and Postpartum: A Practice-Based Perspective. The DEPRESSD Project. Available: http://www.depressionscreening100.com/epds/

(10) Thombs BD, Straus SE, Moore AE. Update on task force terminology and outreach activities. Can Fam Phys 2019; 65(1): 12-13.

(11) Hirschfeld RM, Weissman M. "Risk factor for major depression and bipolar disorder," in neuropsychopharmacology: the fifth generation of progress. p. 1017–25.

(12) Public Health Agency of Canada. Family-Centred Maternity and Newborn Care: National Guidelines. December 2017; Available at: https://www.canada.ca/en/public-health/services/maternity-newborn-care-guidelines.html.

Appendix 1, as supplied by the authors. Appendix to: Lang E, Colquhoun H, LeBlanc JC, et al; Canadian Task Force on Preventive Health Care Recommendation on instrument-based screening for depression during pregnancy and the postpartum period. *CMAJ* 2022. doi: 10.1503/cmaj.220290. Copyright © 2022 The Author(s) or their employer(s). To receive this resource in an accessible format, please contact us at cmajgroup@cmaj.ca.
