# Supplementary material for: Recommendation on instrument-based screening for depression during pregnancy and the postpartum period
Source: CMAJ. 2022 Jul 25;194(28):E981–9. doi: 10.1503/cmaj.220290 (PMC9328462; doi:10.1503/cmaj.220290)
Supplement: Appendix 2. Summary of disclosures of interest for this guideline [file 220290-guide-2-at.pdf]

Appendix 2. Summary of disclosures of interest for this guideline

Canadian Task Force on Preventive Health Care members:

Conflict of interest statements for all task force are made available publicly on the task force website [https://canadiantaskforce.ca/about/members/]. Brett D. Thombs, declared an intellectual interest related to his funded research program and publications on the subject of depression screening. He was a content expert for this guideline; He did not participate in discussions on the recommendation and did not provide input on the direction or strength or vote on the recommendation. Michael Kidd declared an intellectual interest related to funding for research related to depression (National Health and Medical Research Council, Australia) and his role as a Director at Beyond Blue. He was a member of the task force during the early development of this guideline, but he was not a member of the working group and did not provide input or vote on the final recommendation. He was also not an author or contributor to this guideline. Ahmed Abou-Setta, who was a voting task force member, but not a member of the topic working group, reported that he was the editor of the journal 'Systematic Reviews' at the time of publication of the protocol for the systematic review that informed this guideline in this journal. This was not deemed to represent a conflict of interest, and therefore no action was warranted or taken. Scott Klarenbach, who was a task force member but not a member of the topic working group, is the Director of the Real World Evidence Unit, University of Alberta and Director and Co-Chair of the Real World Evidence Consortium (with University of Calgary and Institute of Health Economics). Although he receives no personal honoraria, the group relies in part on industry funding. This was not judged as a conflict when initially disclosed. However, between the initial submission of this guideline to the journal and revising the guideline following peer review, the decision was made that he should not vote on the revised guideline. He did not vote on any changes made to the guideline in response to peer review, or approve resubmission, and as such is not listed as a contributing author. Emily G. McDonald, who was a voting task force member, but not a member of the topic working group, is a co-owner of MedSafer. MedSafer is a software that guides prescribers through the process of deprescribing; it was developed with funding from public granting agencies. This was not deemed to represent a conflict of interest, and therefore no action was warranted or taken. All other task force members declared no interests relevant to this guideline.

Clinical and content experts:

Clinical and content experts to the working group are also required to disclose relevant interests at the outset of their participation and annually thereafter. No relevant interests were declared by Dr. Patten. Dr. Lauria-Horner disclosed receiving payment for an advisory role at the Mental Health Commission of Canada. Dr. Vigod disclosed that she received funding for research activities related to mental health during pregnancy and the postpartum period. Dr. Vigod was also a member of the executive committee for the Women's Mental Health section of the World Psychiatric association and the International Association of Women's Mental Health (2015-2017; unpaid). Dr. Vigod also disclosed receiving funds for an advisory role at UpToDate related to mental health and treatment during pregnancy and postpartum. The task force determined that none of these disclosures represented conflicts of interest that precluded participation as clinical or content experts. Dr. Thombs is a member of the Task Force but acted as a content expert for this guideline as described above.

Appendix 2, as supplied by the authors. Appendix to: Lang E, Colquhoun H, LeBlanc JC, et al; Canadian Task Force on Preventive Health Care Recommendation on instrument-based screening for depression during pregnancy and the postpartum period. *CMAJ* 2022. doi: 10.1503/cmaj.220290. Copyright © 2022 The Author(s) or their employer(s). To receive this resource in an accessible format, please contact us at cmajgroup@cmaj.ca.
