# Supplementary material for: Recommendation on instrument-based screening for depression during pregnancy and the postpartum period
Source: CMAJ. 2022 Jul 25;194(28):E981–9. doi: 10.1503/cmaj.220290 (PMC9328462; doi:10.1503/cmaj.220290)
Supplement: Appendix 3 [file 220290-guide-3-at.pdf]

Appendix 3a. Overview of Guidance for Depression Assessment in Pregnancy or the Prenatal Period by Province/Territory*

	BC	AB	SK	MB	ON	QC	NB	NL	NS	PE	NU	NWT	YK
Ask about past history/ predisposing risk factors for depression	✓1,2ǂ	✓3,4 ǂ	✓5ǂ	✓ 6,7,8ǂ	✓9,10ǂ	✓11ǂ	✓12,13ǂ		✓14,15ǂ	✓16,17,18	✓20ǂ	✓21,34ǂ	
Ask about current anxiety, depression, and/or mood	✓1,2ǂ	✓3	✓5ǂ	✓ 6,8ǂ	✓9,10ǂ		✓12,13ǂ		✓14,15ǂ	✓16, 17,18	✓20ǂ	✓21,34ǂ	
Administer EPDS	✓2, 22-25ǂ		✓5,26,27ǂ		✓9,10ǂ	✓28	✓12,13ǂ			✓30	✓20,29ǂ	✓21,34ǂ	
Administer other instrument (specified or not)		✓3ǂǂ		✓8ǂ*	✓9,10ǂ**					✓19			
Conduct screening using instrument with cut-off score and follow-up action	✓2,24ǂ	✓3ǂǂ	✓5,26,27ǂ		✓9,10ǂ**	✓28	✓12,13ǂ				✓20ǂ	✓21,34ǂ	

* Based on publicly available documentation such as best practice recommendations, care pathways, perinatal records

ǂ Asked in prenatal record

ǂǂ Part of mini-screen in prenatal record; includes both the PHQ-2 and the GAD-2

* Perceived Stress Scale, not universal, part of prenatal record

** PHQ-2; EPDS or PHQ-9 as a follow up to high PHQ-2 score

Appendix 3b. Overview of Guidance for Depression Assessment in the Postnatal Period by Province/Territory*

	BC	AB	SK	MB	ON	QC	NB	NL	NS	PE	NU	NWT	YK
Ask about past history/ predisposing risk factors for depression	✓31ǂ	✓4,32							✓36				
Ask about current anxiety, depression, and/or mood	✓31ǂ	✓4,32	✓26,27	✓6	✓9,10ǂ				✓36		✓39,40		
Administer EPDS	✓24,31ǂ	✓32	✓26,27		✓9,10ǂ	✓28,35	✓12		✓36	✓30	✓39,40	✓21	
Administer other instrument (specified or not)				✓6,37*	✓9,10ǂ**	✓28,35			✓33,36				
Support for depression (not otherwise specified)													✓38
Conduct screening using tool with cut-off score and follow-up action	✓24	✓41	✓26,27		✓9,10ǂ**	✓28,35	✓12		✓33		✓40	✓21	

* Based on publicly available documentation such as best practice recommendations, care pathways, perinatal records

ǂ In postnatal record

*Whooley questions

** PHQ-2; EPDS or PHQ-9 as a follow up to high PHQ-2 score

Note: Shaded cells indicates that no resources were available.

References for Appendix 3

1. Perinatal Services BC. (2014). Perinatal Services BC Provincial Perinatal Guidelines, Population and Public Health Prenatal Care Pathway. Retrieved February 19, 2020, from http://www.perinatalservicesbc.ca/Documents/Guidelines-Standards/HealthPromotion/PrenatalCarePathway.pdf
2. Perinatal Services BC. (2011). British Columbia Antenatal Record Part 2. Retrieved February 24, 2020, from http://www.perinatalservicesbc.ca/Documents/Form/Form1582_AntenatalRecord1and2.pdf
3. Maternal Newborn Child & Youth SCN. (2019). Alberta Antenatal Pathway [Version 1.3, November 15 2019]. Alberta Health Services. Retrieved from https://www.albertahealthservices.ca/assets/about/scn/ahs-scn-mncy-antenatal-pathway.pdf
4. Government of Alberta (2012). Alberta Prenatal Record. Retrieved May 15, 2020, from https://aphp.dapasoft.com/PublicHTML/doc/AB%20Prenatal%20Rec%20HS0001-125.pdf

Appendix 3, as supplied by the authors. Appendix to: Lang E, Colquhoun H, LeBlanc JC, et al; Canadian Task Force on Preventive Health Care Recommendation on instrument-based screening for depression during pregnancy and the postpartum period. *CMAJ* 2022. doi: 10.1503/cmaj.220290. Copyright © 2022 The Author(s) or their employer(s). To receive this resource in an accessible format, please contact us at cmajgroup@cmaj.ca.

5. Saskatchewan Prenatal Record. (2019). Retrieved February 19, 2020, from https://www.ehealthsask.ca/services/resources/Resources/Prenatal-Record-Form-2019.pdf
6. Winnipeg Regional Health Authority. (2016). Perinatal Mental Health Perinatal Quick Reference for Health-Care and Social Service Providers. Retrieved May 15, 2020, from https://professionals.wrha.mb.ca/old/extranet/publichealth/files/PerinatalGuide.pdf
7. Government of Manitoba. (2019). Public Health Nursing Prenatal Practice: Evidence Informed Care Pathway. Retrieved February 19, 2020 from https://professionals.wrha.mb.ca/old/extranet/publichealth/files/PMHToolkitDEC2014.pdf
8. Manitoba Prenatal Record. (n.d.). Retrieved February 19, 2020, from https://www.gov.mb.ca/health/primarycare/providers/docs/prenatalrecordform.pdf
9. Provincial Council for Maternal and Child Health (PCMCH) and The Better Outcomes Registry & Network (BORN) Ontario Perinatal Record Working Group. (2018, August). A User Guide to the Ontario Perinatal Record. Retrieved February 19, 2020, from https://www.pcmch.on.ca/wp-content/uploads/2018/08/OPR_UserGuide_2018Update_Final_18-08-22.pdf
10. Ontario Ministry of Health and Long-Term Care. (2017). Ontario Perinatal Record. Retrieved May 15, 2020 from https://www.pcmch.on.ca/wp-content/uploads/2017/06/OPR-2017.pdf
11. Government of Quebec. (2016, October). Obstetrical File Medical Observations. Retrieved February 19, 2020, from http://msssa4.msss.gouv.qc.ca/intra/formres.nsf/c6dfb077f4130b4985256e38006a9ef0/35eade3927cf912285256ed60044f9cc/$FILE/AH-266A_DT9036(2016-10)D.pdf
12. Horizon Health Network, & Vitality Health Network. (2015). The New-Brunswick Perinatal Health Program: A Completion Guide of the Antenatal Record. Retrieved February 19, 2020, from https://en.horizonnb.ca/media/755769/completion_guide_-_antenatal_record.pdf
13. PérinatalNB. (2018). PérinatalNB Antenatal Record (Part 2). Retrieved May 20, 2020, from https://www.horizonnb.ca/media/755777/hhn-0924_antenatal_record_pt_2.pdf
14. Reproductive Care Program of Nova Scotia. (2015). Nova Scotia Prenatal Record Companion Document. Retrieved February 19, 2020, from http://rcp.nshealth.ca/sites/default/files/publications/PNR Companion Document July 2015.pdf
15. Reproductive Care Program of Nova Scotia. (2015). Nova Scotia Prenatal Record 1. Retrieved May 20, 2020, from http://rcp.nshealth.ca/sites/default/files/chartforms/pnr01_201503.pdf
16. PEI Reproductive Care Program. (1998). Prenatal Psychosocial Health Assessment. Retrieved February 19, 2020, from http://www.gov.pe.ca/photos/original/doh_pnpsychos2.pdf
17. PEI Reproductive Care Program, & Health PEI. (2015). At-a-Glance: Guidelines for Prenatal Laboratory Screening and Testing. Retrieved February 19, 2020, from https://src.healthpei.ca/sites/src.healthpei.ca/files/At_a_Glance_Guidelines_for_Prenatal_Laboratory_Screening_and_Testing.pdf
18. PEI Reproductive Care Program, & Midmer, D. (1999, October). Prenatal Psychosocial Health Assessment Reference Guide. Retrieved February 19, 2020, from https://src.healthpei.ca/sites/src.healthpei.ca/files/Prenatal_Psychosocial_Health_Assessment_Guide.pdf
19. PEI Reproductive Care Program. (1999). Ask Me! Identifying Stressors for Pregnant Women. Retrieved May 20, 2020, from https://src.healthpei.ca/sites/src.healthpei.ca/files/Ask_Me_Identifying_Stressors_for_Pregnant_Women.pdf
20. Government of Nunavut, Department of Health. (2016). Guidelines for Completing Prenatal Record. Retrieved February 19, 2020, from https://www.gov.nu.ca/sites/default/files/guidelines_for_completing_prenatal_record_april_2016_2.pdf
21. Government of Northwest Territories. (2017). A User's Guide for Completion of the NWT Prenatal Record, 2017. Retrieved February 19, 2020, from https://www.hss.gov.nt.ca/professionals/sites/professionals/files/resources/prenatal-record-users-guide.pdf
22. BC Reproductive Mental Health Program and Perinatal Services BC. (2014). Edinburgh Perinatal/Postnatal Depression Scale (EPDS) Scoring Guide. Retrieved February 19, 2020, from http://www.perinatalservicesbc.ca/Documents/Resources/HealthPromotion/EPDS/EPDSScoringGuide_March2015.pdf
23. BC Perinatal Health Program. (2010, February). BCPHP Obstetric Guideline 19: Maternity Care Pathway. Retrieved February 19, 2020, from http://www.perinatalservicesbc.ca/Documents/Guidelines-Standards/Maternal/MaternityCarePathway.pdf
24. BC Reproductive Mental Health Program, BC Women's Hospital & Health Centre, an Agency of the Provincial Health Services Authority, BC Ministry of Health, & Mental Health and Addictions Branch and Healthy Children, Women and Seniors Branch. (2006). Addressing Perinatal Depression: A Framework for BC's Health Authorities. Retrieved February 19, 2020, from http://www.health.gov.bc.ca/library/publications/year/2006/MHA_PerinatalDepression.pdf
25. Perinatal Services BC. (2012). Perinatal Services BC A Guide for Completion of the Antenatal Record Part 1 and 2. Retrieved February 24, 2020, from www.perinatalservicesbc.ca/Documents/Form/Form1582_Guide_AntenatalRecord1and2.pdf
26. Saskatchewan Prevention Institute. (2012). Edinburgh Postpartum Depression Scale (EPDS) Screening and Care Guide: Guide and Manual. Retrieved February 19, 2020, from https://skprevention.ca/resource-catalogue/mental-health/epds-screening-and-care-guide/

 Copyright © 2022 The Author(s) or their employer(s). To receive this resource in an accessible format, please contact us at cmajgroup@cmaj.ca.

27. University of Saskatchewan, Saskatchewan Public Health Association, Saskatchewan Prevention Institute, Canadian Institutes for Health Research, Saskatchewan Psychiatric Association, & Saskatchewan Health Authority. (2019). Maternal Mental Health: Edinburgh Postnatal Depression Scale. Retrieved February 19, 2020, from https://www.ehealthsask.ca/services/resources/Resources/EPDS-screening-2019.pdf
28. Institut national de santé publique du Québec. (2019). Santé mentale et troubles mentaux, Portail d'information périnatale. Retrieved February 24, 2020, from https://www.inspq.qc.ca/sites/default/files/documents/information-perinatale/sante-mentale.pdf
29. Government of Canada. (2011). Adult Care - Chapter 15 - Mental Health. Retrieved February 24, 2020, from https://www.canada.ca/en/indigenous-services-canada/services/first-nations-inuit-health/health-care-services/nursing/clinical-practice-guidelines-nurses-primary-care/adult-care/chapter-15-mental-health.html
30. Government of Prince Edward Island (2018). Pregnancy and Postpartum (Perinatal) Mood Disorders. Retrieved May 21, 2020, from https://www.princeedwardisland.ca/en/information/health-pei/pregnancy-and-postpartum-perinatal-mood-disorders
31. Perinatal Services BC. (2011). British Columbia Community Postpartum Assessment. Retrieved February 24, 2020, from http://www.perinatalservicesbc.ca/Documents/Form/Form1596_CommunityPostpartumAssessment.pdf
32. Maternal Newborn Child & Youth Strategic Clinical Network. (2019). Alberta Pregnancy Pathways. Retrieved February 24, 2020, from https://www.albertahealthservices.ca/assets/about/scn/ahs-scn-mncy-pp-nb-pathway.pdf
33. MacDonald, Joanne and Flynn, Coleen. (2012). Mothers' Mental Health Toolkit, A Resource for the Community. Retrieved February 24, 2020, from http://www.iwk.nshealth.ca/themes/iwkhc/downloads/mmh-toolkit.pdf
34. Government of Northwest Territories. (n.d.). NWT Prenatal Record – Part 1 to Part 6. Retrieved May 21, 2020, from https://www.hss.gov.nt.ca/professionals/sites/professionals/files/resources/nwt-prenatal-record.pdf
35. Fournier, L., Roberge, P., Brouillet, H. (2012). Faire face à la dépression au Québec. Protocole de soins à l'intention des intervenants de première ligne. Montréal : Centre de recherche du CHUM. Retrieved February 24, 2020, from https://www.inspq.qc.ca/pdf/publications/1509_FaireFaceDepressionQc_ProtoSoinsInterv1reLigne.pdf
36. Reproductive Care Program of Nova Scotia. (n.d.). Healthy Babies, Healthy Families: Postpartum & Postnatal Guidelines. Retrieved February 24, 2020 from http://rcp.nshealth.ca/sites/default/files/publications/healthy_babies_healthy_families.pdf
37. Healthy Child Manitoba. (2017, May). Healthy Baby Community Program Guide. Retrieved February 24, 2020 from https://www.gov.mb.ca/healthychild/healthybaby/hb_programguide.pdf
38. Government of Yukon. (2018, June 26). Prenatal and postpartum support. Retrieved February 24, 2020, from http://www.hss.gov.yk.ca/prenatalpostpartum.php
39. Government of Nunavut. (2016). Well Child Record. Retrieved May 21, 2020 from https://www.gov.nu.ca/sites/default/files/well_child_record_nunavut_2016_may_16_2016.pdf
40. Government of Nunavut. (2016). Guidelines for Completing Well Child Record. Retrieved May 21, 2020 from https://www.gov.nu.ca/sites/default/files/files/guidelines_for_completing_well_child_record_2016.pdf
41. Alberta Health Service. (2019, March 6). Postpartum Depression Screening. Retrieved June 5, 2020 from https://extranet.ahsnet.ca/teams/policydocuments/1/clp-prov-public-health-well-child-ppd-screen-guideline-hcs-229-01.pdf

 Copyright © 2022 The Author(s) or their employer(s). To receive this resource in an accessible format, please contact us at cmajgroup@cmaj.ca.
