# Supplementary material for: Recommendation on instrument-based screening for depression during pregnancy and the postpartum period
Source: CMAJ. 2022 Jul 25;194(28):E981–9. doi: 10.1503/cmaj.220290 (PMC9328462; doi:10.1503/cmaj.220290)
Supplement: Appendix 4. Asesing Depresion During the Pregnancy and Postpartum Period by Province/Territory [file 220290-guide-4-at.pdf]

Appendix 4. Assessing Depression During the Pregnancy and Postpartum Period by Province/Territory

Alberta		
Prenatal	**Past history/ predisposing factors**	• At week 10 or initial booking appointment: Alberta Antenatal pathway suggests that patients be "ask[ed] about anxiety/depression and any past or present mental illness or psychiatric treatment" by primary care physician/midwife. [1]
	**Asking about current anxiety, depression, and/or mood**	• PCN nurse/Clinic nurse to provide counselling on various topics, including "mental health" and at week 10/initial booking appointment; at weeks 21-27, 28-32. [1]<br>• PCN nurse/Clinic nurse to provide counselling on "post-partum depression" at weeks 40-42. [1]<br>• Primary care physicians/midwives "ask about anxiety/depression" at weeks 16, 28. [1]<br>• Primary care physicians/midwives "assess for anxiety/depression" at weeks 20, 24, 34, 36. [1]
	**Screening**	• Universal perinatal mental health screening is recommended on the basis of its high benefit and low harm. [1]
	**Use of EPDS**	Not applicable
	**Other Tool(s)**	• Complete the mini screen for prenatal anxiety or depression within the 1st, 2nd and 3rd trimesters, to assess risk and need for referral to mental health assessment program. Use of mini-screen located in prenatal record; comprised of PHQ-2 and GAD-2 (for anxiety). [1]
Postnatal	**Past history/ predisposing factors**	• Alberta "Initial Postpartum and Newborn Care" pathway for nurses includes a section on Psychosocial Health: Emotional and Mental Health Assessment during post-partum period (ranging from 0-2 hours to >72 hours-7 days [and beyond]). [2]<br>• Assessment includes assessing the "…Emotional response to delivery and postpartum period (current & **past** *[emphasis added]*)," "predisposing risk factors for postpartum depression (PPD such as previous episodes of depression, family history of depression, previous use of anti-depressants, significant obstetrical or medical challenges," and "signs of PPD". [2]
	**Asking about current anxiety, depression, and/or mood**	• Psychosocial Health: Emotional and Mental Health Assessment during post-partum period (ranging from 0-2 hours to >72 hours-7 days [and beyond]) includes assessing the "emotional state" and "emotional response to delivery and postpartum period (**current** *[emphasis added]* and past)". [2]<br>• Also captured in Postpartum Clinical Documentation and Postpartum Clinical Path as Emotional and Mental Health (section on Mothering) [2]
	**Use of EPDS**	• At 2 months post-partum, EPDS to be used for "screening and education" by Alberta Health Services public health staff. [3]<br>○ Score of 10-12: Likelihood of depression considered moderate, discuss, inform of resources, follow-up based on clinical judgement.<br>○ Score of 12 or more: likelihood of depression is considered high, discuss, offer referral, develop a Family Support Plan, inform of services, follow-up based on clinical judgement.<br>○ Positive score on question 10 (suicidality risk): Use Suicide Risk flowchart, follow-up based on clinical judgement
	**Other Tool(s)**	Not applicable

[1] Alberta Health Services. Alberta Antenatal Pathway. Version 1.3. Edmonton: Alberta Health Services, 2019. [Accessed 2019-02-15] https://www.albertahealthservices.ca/assets/about/scn/ahs-scn-mncy-antenatal-pathway.pdf

Appendix 4, as supplied by the authors. Appendix to: Lang E, Colquhoun H, LeBlanc JC, et al; Canadian Task Force on Preventive Health Care Recommendation on instrument-based screening for depression during pregnancy and the postpartum period. *CMAJ* 2022. doi: 10.1503/cmaj.220290. Copyright © 2022 The Author(s) or their employer(s). To receive this resource in an accessible format, please contact us at cmajgroup@cmaj.ca.

[2] Alberta Health Services, Covenant Health. Alberta Pregnancy Pathways: Initial Postpartum & Newborn Care. Version 4.1. Edmonton: Alberta Health Services, 2019. [Accessed 2020-02-15] https://www.albertahealthservices.ca/assets/about/scn/ahs-scn-mncy-pp-nb-pathway.pdf

3 Alberta Health Services. Postpartum Depression Screening. Edmonton: Alberta Health Services, 2019. [Accessed 2020-08-13] https://extranet.ahsnet.ca/teams/policydocuments/1/clp-prov-public-health-well-child-ppd-screen-guideline-hcs-229-01.pdf.

British Columbia		
Prenatal	**Past history/ predisposing factors**	• Section 6 of the BC Antenatal Record ("Family History") includes a check box and comment field to capture any information on a "Family History."[1] Follow-up check boxes for specific diagnoses (including depression/psychiatric) are also found in this section.[1]<br>• Section 7 of the BC Antenatal Record ("Medical History") includes a check box and comment field to capture any information on a "history of medical illness."[1] Follow-up check boxes for specific diagnoses (including anxiety, depression, bipolar, PP depression, unknown, other) are also found in this section. [1]<br>• The BC "Population and Public Health Prenatal Care Pathway" includes a section on "Emotional Health and Adjustment to Pregnancy" that highlights that patients with a "…history of depression (previous history of PND, personal or family history of mental health challenges or disorders such as depression, anxiety, bipolar or psychotic disorders" may "require support or medical assessment".[2]
	**Asking about current anxiety, depression, and/or mood**	• The BC "Population and Public Health Prenatal Care Pathway" includes a section on "Emotional Health and Adjustment to Pregnancy" that recommends assessment of a woman's emotional response and adjustment to her pregnancy and her integration of her role of becoming a mother [2]
	**Use of EPDS**	• The BC "Population and Public Health Prenatal Care Pathway," [2] the "BC Maternity Care Pathway"[3] and the "BC Health Authorities Perinatal Depression Framework"[4] recommend that the EPDS be administered to all patients between 28-32 weeks.<br>• The EPDS tool and score guidelines are reported in the "Edinburgh Perinatal/Postnatal Depression Scale (EPDS) Scoring Guide."[5]<br>○ Score of 9-11: Depression possible. Support, rescreen in 2-4 weeks.<br>○ Score of 12-13: Fairly high possibility of depression: Monitor, support, offer education. Refer to primary care provider.<br>○ Score of 14 or above: Probable depression: diagnostic assessment and treatment.<br>○ Positive score on question 10 (suicidality risk): Immediate discussion and referral as required
	**Other Tool(s)**	Not specified
Postnatal	**Past history/ predisposing factors**	• BC's Community Postpartum Assessment includes a "Psychosocial" assessment section, it asks about history of postpartum depression and/or other mental illness. [6]<br>• Unclear who is responsible for administering this assessment.
	**Asking about current anxiety, depression, and/or mood**	• BC's Community Postpartum Assessment "Psychosocial" assessment section also asks about "emotional adjustment". [6]<br>• Unclear who is responsible for administering this assessment.
	**Use of EPDS**	• BC's Community Postpartum Assessment "Psychosocial" assessment section also includes a section to capture the postpartum EPDS score, with scoring noted above.[6]<br>• This is consistent with the guidance provided within the "BC Health Authorities Perinatal Depression Framework," whereby the EPDS is to be administered 6-8 weeks postpartum.[4]

 Copyright © 2022 The Author(s) or their employer(s). To receive this resource in an accessible format, please contact us at cmajgroup@cmaj.ca.

	**Other Tool(s)**	Not specified

[1] Perinatal Services BC. British Columbia Antenatal Record. Vancouver: Perinatal Services BC, 2011. [Accessed 2020-02-17] http://www.perinatalservicesbc.ca/Documents/Form/Form1582_AntenatalRecord1and2.pdf
[2] Perinatal Services BC. Provincial Perinatal Guidelines. Population and Public Health Prenatal Care Pathway. Vancouver: Perinatal Services BC, 2014. [Accessed 2020-02-17] http://www.perinatalservicesbc.ca/Documents/Guidelines-Standards/HealthPromotion/PrenatalCarePathway.pdf
[3] BC Perinatal Health Program. BCPHP Obstetric Guideline 19: Maternity Care Pathway. Vancouver: BC Perinatal Health Program, 2010. [Accessed 2020-02-17] http://www.perinatalservicesbc.ca/Documents/Guidelines-Standards/Maternal/MaternityCarePathway.pdf
[4] BC Reproductive Mental Health Program. Addressing Perinatal Depression: A Framework for BC's Health Authorities. Vancouver: BC Reproductive Mental Health Program, 2006. [Accessed 2020-02-17] http://www.health.gov.bc.ca/library/publications/year/2006/MHA_PerinatalDepression.pdf
[5] BC Reproductive Mental Health Program and Perinatal Services BC. Edinburgh Perinatal/Postnatal Depression Scale (EPDS) Scoring Guide. Vancouver: Perinatal Services BC, 2014. [Accessed 2020-02-19] http://www.perinatalservicesbc.ca/Documents/Resources/HealthPromotion/EPDS/EPDSScoringGuide_March2015.pdf
[5] Perinatal Services BC. British Columbia Community Postpartum Assessment. Form PSBC1596. Vancouver: Perinatal Services BC, 2011. [Accessed 2020-02-17] http://www.perinatalservicesbc.ca/Documents/Form/Form1596_CommunityPostpartumAssessment.pdf

Saskatchewan		
Prenatal	**Past history/ predisposing factors**[1]	• The "Health History" section of the Saskatchewan prenatal record form includes a check box for anxiety/depression. The form also includes an "additional comments" section where the individual completing the form can "provide details and any referrals."<br>• It is unclear who is responsible for administering the prenatal record.
	**Asking about current anxiety, depression, and/or mood**[1]	• "Mental health" is included in the prenatal record as a discussion topic during all three trimesters. This provides an opportunity to the individual completing the form to ask about current anxiety, depression and/or mood.
	**Use of EPDS**	• Saskatchewan recommends that the EPDS be administered twice at minimum – once at the first prenatal visit, and once at 28-21 weeks gestation, or as deemed necessary by the practitioner.[2]<br>• The Saskatchewan prenatal record includes a section where the EPDS score should be captured[1] and scoring instructions are as follows[3]:<br>o Score of 10-11: Possible depression.<br>o Score of 12 or above: probable depression. Offer referral to family doctor or nurse practitioner.<br>o Positive score on question 10 (suicidality risk): Assess harm intentions and for psychosis<br>• As with the SK prenatal record, it is unclear who is mandated to administer the EPDS.
	**Other Tool(s)**	Not applicable
Postnatal	**Past history/ predisposing factors**	Not applicable
	**Asking about current anxiety,**	Not applicable

 Copyright © 2022 The Author(s) or their employer(s). To receive this resource in an accessible format, please contact us at cmajgroup@cmaj.ca.

	**depression, and/or mood**	
	**Use of EPDS**	• The "EPDS Screening and Care Guide," which is the basis for the perinatal EPDS screening, also recommends that the EPDS be administered at 2-3 weeks postpartum, at 2-month, and at 6-month well child visits. [2]<br>• It can also be administered "as deemed necessary by the practitioner" [2]
	**Other Tool(s)**	Not applicable

[1] Saskatchewan Health. Saskatchewan Prenatal Record. Form H19-42 (03/19) PRF. [Accessed 2020-02-18] https://www.ehealthsask.ca/services/resources/Resources/Prenatal-Record-Form-2019.pdf
[2] Saskatchewan Prevention Institute. Edinburgh Postpartum Depression Scale (EPDS) Screening and Care Guide: Guide and Manual, 2012. Regina: Saskatchewan Prevention Institute, 2012. [Accessed 2020-02-18] https://skprevention.ca/resource-catalogue/mental-health/epds-screening-and-care-guide/
[3] Saskatchewan Maternal Mental Health. Maternal Mental Health: EPDS Screening and Care Guide. 2020. [Accessed 2020-08-13] https://skmaternalmentalhealthca.files.wordpress.com/2018/09/edinburgh-postnatal-depression-screen-and-care-guide.pdf

Manitoba

Prenatal	**Past history/ predisposing factors**	• The Winnipeg Regional Health Authority "Perinatal Mental Health" reference guide for "Health-Care and Social Service Providers" encourages providers to ask pregnant patients about their mental health history.[1]<br>• Specific questions as part of the "questions to ask prenatal women" include "have you had emotional problems?" "Have you ever seen a psychiatrist, psychologist, or therapist?" [1]<br>• MB public health nurses can also conduct prenatal psychosocial assessments, where they may broach questions such as "is there a history of mental health issues in your family?" "Have you ever suffered from depression, anxiety, or psychosis? If so, when did it occur and what did you do about it?"[2]<br>• These practices are consistent with the MB "Public Health Nursing Prenatal Practice: Evidence Informed Care Pathway" that states that a nurse role includes assessing "for predisposing or risk factors to perinatal depression such as a previous history of depression/anxiety, family history of depression/anxiety, previous use of antidepressants […]" (Section 5). [3]<br>• The Manitoba Prenatal Record form includes a "Family History" section, with check boxes for a number of conditions as well as an area for comments. Of relevance are the check boxes for "mental illness" and "depression".[4]
	**Asking about current anxiety, depression, and/or mood**	• The Manitoba "Perinatal Mental Health" reference guide for "Health-Care and Social Service Providers" encourages providers to ask pregnant patients about their mood ("how has your mood been during this pregnancy?") [1]<br>• As part of the prenatal psychosocial assessment, public health nurses may also as probing questions about current depression, including "in the past two weeks have you been bothered by feeling down, depressed or hopeless?" "In the past two weeks have you been bothered by having little interest or pleasuring in doing things?" [2]<br>• These practices are also consistent with the MB "Public Health Nursing Prenatal Practice: Evidence Informed Care Pathway" that states that a nurse role includes assessing "the client's emotional response and adjustment to the pregnancy and becoming a parent," (Section 5) [3].<br>• The pathway also states that nurses should "assess for… current signs of depression". [3]

 Copyright © 2022 The Author(s) or their employer(s). To receive this resource in an accessible format, please contact us at cmajgroup@cmaj.ca.

		• The Manitoba Prenatal Record form includes a "Significant Medical Illnesses" section, with check boxes for a number of conditions, as well as an area for comments. Of relevance are the check boxes for "mental illness" and "depression".[4]<br>• The Manitoba Prenatal Record form also includes a "Risk Factors" section, where the individual completing the form can identify "depression" as one of the "lifestyle/social issues" that the feel is relevant to the pregnant woman being evaluated.[4]
	**Use of EPDS**	Not applicable
	**Other Tool(s)**	• The Manitoba Prenatal Record form also includes the "Perceived Stress Scale", which can be used as part of a clinician's evaluation of depressive and other risks in a pregnant woman.<br>• There is no guidance on how to use this tool – no indication of how and when to use, nor how to interpret its findings.[4]
Postnatal	**Past history/ predisposing factors**	Not applicable
	**Asking about current anxiety, depression, and/or mood**	• The Manitoba "Perinatal Mental Health" reference guide for "Health-Care and Social Service Providers" encourages providers to ask postpartum patients a number of mental health-related assessment questions, most adapted from other tools/sources. These include questions about "feeling down, depressed or hopeless"[1]
	**Use of EPDS**	Not applicable
	**Other Tool(s)**	Not applicable

[1] Winnipeg Regional Health Authority. Perinatal Mental Health: Quick Reference for Health-Care and Social Service Providers. Winnipeg: WRHA, 2016. [Accessed 2020-02-18] http://www.wrha.mb.ca/healthinfo/prohealth/files/PerinatalMHGuide%20(2).pdf
[2] Winnipeg Regional Health Authority. Perinatal Mental Health Toolkit. Winnipeg: WHRA, 2014. [Accessed 2020-02-18] http://www.wrha.mb.ca/extranet/publichealth/files/PMHToolkitDEC2014.pdf
[3] Province of Manitoba. Public Health Nursing Prenatal Practice: Evidence Informed Care Pathway. Provincial Standards for Postpartum and Early Childhood: Province of Manitoba. Winnipeg: Province of Manitoba, 2019. [Accessed 2020-02-23] https://www.gov.mb.ca/health/publichealth/phnursingstandards/docs/Prenatal_Evidence_Informed_Care_Pathway.pdf
[4] Manitoba Health. Manitoba Prenatal Record. Form 0600. Winnipeg: Manitoba Health, 2000. [Accessed 2020-02-18] https://www.gov.mb.ca/health/primarycare/providers/docs/prenatalrecordform.pdf

**Ontario**		
Prenatal	**Past history/ predisposing factors**	• Item 25 in the "Family Screen" section of the Ontario Perinatal Record recommends "screen[ing] for family history of depression/psychiatric issues […]".[1]<br>• Item 37 in the "Mental Health/Substance Use" section of the Ontario Perinatal Record also states that "**Past history** *[emphasis added]* or current depression should be documented and include treatment/coping strategies." [1]
	**Asking about current anxiety, depression, and/or mood**	• Item 36 in the "Mental Health/Substance Use" section of the Ontario Perinatal Record also states that "Past history or **current anxiety** *[emphasis added]* should be documented and include treatment/coping strategies." [1]

 Copyright © 2022 The Author(s) or their employer(s). To receive this resource in an accessible format, please contact us at cmajgroup@cmaj.ca.

		• Item 37 in the "Mental Health/Substance Use" section of the Ontario Perinatal Record also states that "Past history or **current depression** *[emphasis added]* should be documented and include treatment/coping strategies." [1]
	**Use of EPDS**	• Item 37 in the "Mental Health/Substance Use" section of the Ontario Perinatal Record proposes the EPDS as a follow-up screening tool "[…] if the PHQ2 score indicates risk."
	**Other Tool(s)**	• **Depression screening using the PHQ-2 is outlined in item 37 in the "Mental Health/Substance section"** of the Ontario Perinatal Record. The description states that the tool can be used multiple times during an individual's pregnancy, especially if an individual appears to be at high risk of depression. [1]<br>• A score of 3 or more should trigger a "consideration of further assessment", using the PHQ-9 or EPDS. [1]<br>• Although the PHQ-9 is proposed as a tool for further assessment (as an alternate to the EPDS), no cut-off scores are provided. [1]
Postnatal	**Past history/ predisposing factors**	• In Ontario Perinatal Record Postnatal Visit, "History" section includes emotional wellbeing and Postpartum Depression Screen (EPDS or other)[2]
	**Asking about current anxiety, depression, and/or mood**	• In Ontario Perinatal Record Postnatal Visit, "History" section includes Postpartum Depression Screen (EPDS or other)[2]
	**Use of EPDS**	• In Ontario Perinatal Record Resources Section, Depression Screening is done using PHQ-2. A total score of 3 or more warrants consideration of using the EPDS or PHQ9 for further assessment or additional mental health follow up. [2]<br>o Score of 9-12: monitor, support, and offer education<br>o Score of 13 or above: follow up with comprehensive bio-psychosocial diagnostic assessment for depression<br>o Positive score on question 10 (suicidality risk): immediate assessment and intervention[2]
	**Other Tool(s)**	• In Ontario Perinatal Record Resources Section, Depression Screening is done using PHQ-2. A total score of 3 or more warrants consideration of using the EPDS or Patient Health Questionnaire (PHQ) 9 for further assessment or additional mental health follow up. [2]

[1] Government of Ontario. A User Guide to the Ontario Perinatal Record. Toronto: Government of Ontario, 2018. [Accessed 2020-02-23] https://www.pcmch.on.ca/wp-content/uploads/2018/08/OPR_UserGuide_2018Update_Final_18-08-22.pdf
[2] Ontario Ministry of Health and Long-Term Care. Ontario Perinatal Record. Toronto: Government of Ontario, 2017. [Accessed 2020-08-13] https://www.pcmch.on.ca/wp-content/uploads/2017/06/OPR-2017.pdf

Quebec		
Prenatal	**Past history/ predisposing factors**	• The obstetrical file includes a "family antecedents" section, with a comment box where relevant family antecedents can be documented [1].<br>• The obstetrical file also includes a "personal antecedents" section, with check boxes, including one for "neuropsychiatric disease." For each selected option, the specific condition(s) must be captured [1].
	**Asking about current anxiety, depression, and/or mood**	Not applicable

 Copyright © 2022 The Author(s) or their employer(s). To receive this resource in an accessible format, please contact us at cmajgroup@cmaj.ca.

	**Use of EPDS**	• INSPQ guidance suggests that EPDS should be administered, and that a cut-off score of 13 or more be used to determine who should be referred to specialized care/further assessment. [2,3]<br>However, information on who should administer the EPDS, and when, is lacking.
	**Other Tool(s)**	Not applicable
Postnatal	**Past history/ predisposing factors**	Not applicable
	**Asking about current anxiety, depression, and/or mood**	Not applicable
	**Use of EPDS**	• INSPQ guidance suggests that postpartum screening should be provided. However, although it explicitly suggests EPDS should for the perinatal period, it is not as prescriptive for the postnatal period. [2,3]<br>• This may simply be an interpretation issue related to the documents' wording; however, this is unclear
	**Other Tool(s)**	• Guidance from the INSPQ recommends screening using the Patient Health Questionnaire (PHQ) 2 questions for people at high risk of depression, including those with hormonal changes such as postpartum. If someone answers yes to one of these questions, clinicians should conduct a full evaluation of the person's mental state and functional, interpersonal, or social difficulties [3]

[1] Ministère de la santé et des services sociaux du Québec. Obstetrical File: Medical Observations. Form # AH-266A DT9036 (rev. 2016-10). [accessed 2020-02-17] http://msssa4.msss.gouv.qc.ca/intra/formres.nsf/c6dfb077f4130b4985256e38006a9ef0/35eade3927cf912285256ed60044f9cc/$FILE/AH-266A_DT9036(2016-10)D.pdf
[2] Institut national de santé publique du Québec. Santé mentale et troubles mentaux en périnatalité. [Accessed 2020-02-17] https://www.inspq.qc.ca/information-perinatale/fiches/sante-mentale#risque
[3] Institut national de santé publique du Québec. Santé mentale et troubles mentaux : Portail d'information périnatale. [Accessed 2020-02-17] https://www.inspq.qc.ca/sites/default/files/documents/information-perinatale/sante-mentale.pdf

New Brunswick		
Prenatal	**Past history/ predisposing factors**[1]	• In the New Brunswick Antenatal Record, Section 4 focuses on capturing "Medical History and Family History." This includes a "depression/psychiatric" subcategory where "present or **past** *[emphasis added]* history of mental illness and treatments" can be documented. [1]
	**Asking about current anxiety, depression, and/or mood**[1]	• In the New Brunswick Antenatal Record, Section 4 focuses on capturing "Medical History and Family History." This includes a "depression/psychiatric" subcategory where "**present** *[emphasis added]* or past history of mental illness and treatments" can be documented. [1]
	**Use of EPDS**[1]	• As part of the Antenatal Record, the EPDS is administered between weeks 28-32. Healthcare providers should indicate the score on the EPDS as well as 'yes' or 'no' for a follow-up. [1]<br>• EPDS is self-administered
	**Other Tool(s)**	Not applicable

 Copyright © 2022 The Author(s) or their employer(s). To receive this resource in an accessible format, please contact us at cmajgroup@cmaj.ca.

Postnatal	**Past history/ predisposing factors**	Not applicable
	**Asking about current anxiety, depression, and/or mood**	Not applicable
	**Use of EPDS**[1]	• In section 7 of the antenatal record, the "EPDS" item description includes a recommendation that patients be screened again at 6-8 weeks postpartum, using the same instructions and cut-offs as during the prenatal period. [1]
	**Other Tool(s)**	Not applicable

[1] Horizon Health Network, Vitalité Health Network. The New-Brunswick Perinatal Health Program: A Completion Guide of the Antenatal Record. Working Tool for Healthcare Providers. Moncton, NB: Horizon and Vitalité Health Networks, no date. [Accessed 2020-02-17] https://en.horizonnb.ca/media/755769/completion_guide_-_antenatal_record.pdf

Nova Scotia		
Prenatal	**Past history/ predisposing factors**	• In the Nova Scotia Prenatal Record, Section 6 ("Past Illness") includes "mental health," with an opportunity for the individual completing the form to capture additional comments. [1]
	**Asking about current anxiety, depression, and/or mood**	• In the Nova Scotia Prenatal Record, Section 4 ("Present Pregnancy") provides an "opportunity to gather information about health concerns in present pregnancy including […] **depression**/anxiety [*emphasis added]*". [2]
	**Use of EPDS**	Not applicable
	**Other Tool(s)**	Not applicable
Postnatal	**Past history/ predisposing factors**	• The NS Department of Health "Postpartum & Postnatal Guidelines" includes a section on psychosocial/family adjustments that focuses on "…support[ing] healthy psychosocial adjustment, assessment and intervention strategies." [3]<br>• It emphasizes that "…psychosocial concerns identified in the prenatal period" should be re-assessed at the postnatal period and monitored. [3]
	**Asking about current anxiety, depression, and/or mood**	• The NS Department of Health "Postpartum & Postnatal Guidelines" section on psychosocial/family adjustments also states "all women should be assessed for the risk of, or the presence of postpartum depression…". [3]
	**Use of EPDS**	• The NS Department of Health "Postpartum & Postnatal Guidelines" section on psychosocial/family adjustments also states "A standardized postpartum depression screening tool could be used" for assessing the risk of, or presence of, postpartum depression. The EPDS is included as a depression screening tool. [3] However, no cut-off scores are provided as part of these guidelines.<br>• The "Mothers' Mental Health Toolkit," published by NS Health, states that "if you suspect the woman you are working with may be depressed, you can use [the EPDS] screening test." [4]

 Copyright © 2022 The Author(s) or their employer(s). To receive this resource in an accessible format, please contact us at cmajgroup@cmaj.ca.

		• This guidance is not provided as part of the NS prenatal record process. Furthermore, the toolkit targets a broad population (“women, their family and friends, community service providers”). [4]<br>• The toolkit recommends that “a woman scoring nine or more points [on the EPDS] or indicating any suicidal ideation – that is, she scores 1 or higher on question 10 – should be referred immediately for follow-up. Even if a woman scores less than 9, if the clinician feels the client is suffering from depression, an appropriate referral should be made.” [4]
	**Other Tool(s)**	• In addition to the EPDS, Attachment 12 of the NS Department of Health “Postpartum & Postnatal Guidelines” (“Postpartum Depression Screening Tools”) also lists the Postpartum Depression Screening Scale (PDSS) as a possible screening tool to be used as part of the postpartum assessment strategies. [3]<br>• No cut-off scores are provided to evaluate the PDSS. [3]

[1] Reproductive Care Program of Nova Scotia. Nova Scotia Prenatal Record 1. Halifax: RCP, 2015. [Accessed 2020-02-17] http://rcp.nshealth.ca/sites/default/files/chartforms/pnr01_201503.pdf
[2] Reproductive Care Program of Nova Scotia. Nova Scotia Prenatal Record Companion Document. Revised July 2015. Halifax: RCP, 2015. [Accessed 2020-02-17] http://rcp.nshealth.ca/sites/default/files/publications/PNR%20Companion%20Document%20July%202015.pdf
3 Nova Scotia Department of Health. Health Babies, Healthy Families: Postpartum & Postnatal Guidelines. A report prepared by the Reproductive Care Program of Nova Scotia. Halifax: Nova Scotia Department of Health, 2003. [Accessed 2020-02-17] http://rcp.nshealth.ca/sites/default/files/publications/healthy_babies_healthy_families.pdf
[4] Reproductive Mental Health Service. Mothers’ Mental Health Toolkit: A Resource for the Community. Halifax: IWK Health Centre, 2012. [Accessed 2020-02-17] http://www.iwk.nshealth.ca/themes/iwkhc/downloads/mmh-toolkit.pdf

Nunavut		
Prenatal	**Past history/ predisposing factors**	• Section 6 (“Family History”) of prenatal record includes “family history of… depression…” in the families of either the pregnant woman or the baby’s father. [1]<br>• If “yes” is selected, the person completing the prenatal record is asked to “document/explain”. [1]<br>• Section 7 (“Medical History”) of prenatal record also captures “Past *[emphasis added]* or current history of mental illness. Indicate if history of anxiety, depression/postpartum depression and/or other condition(s) and specify.” [1]<br>• Notes for the subsection on “history of mental illness”, which states that “Particular attention should be paid to history of postpartum depression.” [1]<br>• Prenatal record can be administered by any health care provider. [1]
	**Asking about current anxiety, depression, and/or mood**	• Section 7 (“Medical History”) of prenatal record also captures “Past or **current** *[emphasis added]* history of mental illness. Indicate if history of anxiety, depression/postpartum depression and/or other condition(s) and specify.” [1]
	**Use of EPDS**	• EPDS is included as Part 2B of prenatal record. [2]<br>• To be administered between 28-32 weeks as part of universal screening program. [2]<br>• “Refer women who score 14 or above to mental health services as appropriate. If a woman reports thoughts of self-harm she should be referred to mental health services immediately.” [2]
	**Other Tool(s)**	Not applicable
Postnatal	**Past history/ predisposing factors**	Not applicable

 Copyright © 2022 The Author(s) or their employer(s). To receive this resource in an accessible format, please contact us at cmajgroup@cmaj.ca.

	**Asking about current anxiety, depression, and/or mood**	Not applicable
	**Use of EPDS**	• In section 22 of the prenatal record, the "EPDS" item description includes a recommendation that patients be screened again at 6-8 weeks postpartum, using the same instructions and cut-offs as during the perinatal period. [2]
	**Other Tool(s)**	Not applicable

[1] Nunavut Department of Health. Guidelines for Completing Prenatal Record. Iqaluit: Nunavut Department of Health, 2016. [Accessed 2020-02-17] https://www.gov.nu.ca/sites/default/files/guidelines_for_completing_prenatal_record_april_2016_2.pdf
[2] Nunavut Department of Health. Nunavut Prenatal Record Guidelines version 2.0: A Guide for Completion of Nunavut Prenatal Records Part 1, 2 and 3. March 2012. Iqaluit: Nunavut Department of Health, 2012. [Accessed 2020-02-17] https://www.uvic.ca/medsci/assets/docs/arbour/Nunavut%20Prenatal%20Record%20Guidelines%20Version%202%20March%202012.pdf

Northwest Territories

Prenatal	**Past history/ predisposing factors[1]**	• Section 8 of the prenatal record includes a subsection on "past *[emphasis added]* or current history of mental illness. Indicate if history of anxiety, depression/postpartum depression and/or other condition(s) and specify." [1]<br>• Notes for the subsection on "history of mental illness", which states that "Particular attention should be paid to the postpartum period, particularly where there is a history of postpartum depression."<br>• Section 14 also includes a "Risk Factors" subsection where the clinician should "indicate risk factors based on obstetrical, family, medical, lifestyle and social histories, and physical assessments in this box." This includes depression<br>• Administered by "prenatal caregivers".
	**Asking about current anxiety, depression, and/or mood**	• Section 8 of the prenatal record includes a subsection on "past or **current** *[emphasis added]* shistory of mental illness. Indicate if history of anxiety, depression/postpartum depression and/or other condition(s) and specify." [1]
	**Use of EPDS**	• Part 6 of the prenatal record includes the use of the EPDS between 28-32 weeks. [2]<br>• Score of 11 to 13 indicates monitoring, support, and education. [2]<br>• Score of 14 or above indicates follow-up with biopsychosocial diagnostic assessment for depression. A positive score on question 10 (suicidality risk) indicates immediate discussion and referral as required. [2]
	**Other Tool(s)**	Not applicable
Postnatal	**Past history/ predisposing factors**	Not applicable
	**Asking about current anxiety, depression, and/or mood**	Not applicable

 Copyright © 2022 The Author(s) or their employer(s). To receive this resource in an accessible format, please contact us at cmajgroup@cmaj.ca.

	**Use of EPDS**	• In section 14 of the prenatal record, the "EPDS" item description includes a footnote that states that "postnatal depression screening should also be completed at 6 weeks postpartum." [1]
	**Other Tool(s)**	Not applicable

[1] Government of Northwest Territories. A User's Guide for Completion of the NWT Prenatal Record 2017. Yellowknife: Government of Northwest Territories, 2017. [Accessed 2020-02-17] https://www.hss.gov.nt.ca/professionals/sites/professionals/files/resources/prenatal-record-users-guide.pdf
[2] Government of Northwest Territories. NWT Prenatal Record – Part 1 to Part 6. Yellowknife: Government of Northwest Territories, n.d. [Accessed 2020-05-21] https://www.hss.gov.nt.ca/professionals/sites/professionals/files/resources/nwt-prenatal-record.pdf

Prince Edward Island

Prenatal	**Past history/ predisposing factors**	• At weeks 16-20+, Health PEI "Guidelines for Prenatal Laboratory Screening and Testing" recommend "Prenatal Psychosocial Health Assessment".[1]<br>• Prenatal Psychological Health Assessment[2] poses questions pertaining to "history of psychiatric or emotional problems".<br>• A supporting reference guide provides probing questions to better assess "history of psychiatric or emotional problems".[3]<br>• Designed for "health care providers," but it is unclear who is provincially responsible for its administration.
	**Asking about current anxiety, depression, and/or mood**	• At weeks 16-20+, Health PEI "Guidelines for Prenatal Laboratory Screening and Testing" recommend "Prenatal Psychosocial Health Assessment".[1]<br>• Prenatal Psychological Health Assessment[2] poses questions pertaining to "depression in this pregnancy".<br>• A supporting reference guide provides probing questions to better assess "depression in this pregnancy".[3]<br>• Designed for "health care providers," but it is unclear who is provincially responsible for its administration.
	**Use of EPDS**	• Public Health Nurses are available to assist/counsel pregnant patients who have self-identified as "…worried about anxiety or depression" with the completion of the EPDS and the provision of "…information about support/help that is available in [their] community". [5]
	**Other Tool(s)** [4]	• Ask Me! self report, during week 16 visit. [4]<br>• To be offered to all pregnant patients. [4]<br>• Administered by a clinician; unclear whether other health care providers (nurses, midwives, etc.) can also administer it. [4]
Postnatal	**Past history/ predisposing factors**	Not applicable
	**Asking about current anxiety, depression, and/or mood**	Not applicable
	**Use of EPDS**[5]	• Recommended during child's 2-month health clinic visit; it is unclear who administers it at this time. [5]

 Copyright © 2022 The Author(s) or their employer(s). To receive this resource in an accessible format, please contact us at cmajgroup@cmaj.ca.

		• Public Health Nurses are also available to assist/counsel individuals who self-identify as "…concerned about how [they] are feeling" after the birth of their child.
	**Other Tool(s)**	Not applicable

[1] Health PEI. At-a-Glance: Guidelines for Prenatal Laboratory Screening and Testing. [Accessed 2020-02-17] https://src.healthpei.ca/sites/src.healthpei.ca/files/At_a_Glance_Guidelines_for_Prenatal_Laboratory_Screening_and_Testing.pdf
[2] Healthy Mothers, Healthy Babies. Prenatal Psychological Health Assessment. [Accessed 2020-02-17] http://www.gov.pe.ca/photos/original/doh_pnpsychos2.pdf
[3] ALPHA Group. Prenatal Psychological Health Assessment: Reference Guide. Toronto, ON: ALPHA Group, 1999. [Accessed 2020-02-17] https://src.healthpei.ca/sites/src.healthpei.ca/files/Prenatal_Psychosocial_Health_Assessment_Guide.pdf
[4] Health PEI. Prenatal Psychological Assessment Guidelines. [Accessed 2020-02-17] https://src.healthpei.ca/sites/src.healthpei.ca/files/carepathways/hpei_pre_ppag_0.pdf
[5] Health PEI. Pregnancy and Postpartum (Perinatal) Mood Disorders. [Accessed 2020-02-17] https://www.princeedwardisland.ca/en/information/health-pei/pregnancy-and-postpartum-perinatal-mood-disorders

Newfoundland and Labrador

Prenatal	**Past history/ predisposing factors**	Not applicable
	**Asking about current anxiety, depression, and/or mood**	Not applicable
	**Use of EPDS**	Not applicable
	**Other Tool(s)**	Not applicable
Postnatal	**Past history/ predisposing factors**	Not applicable
	**Asking about current anxiety, depression, and/or mood**	Not applicable
	**Use of EPDS**	Not applicable
	**Other Tool(s)**	Not applicable

Yukon

 Copyright © 2022 The Author(s) or their employer(s). To receive this resource in an accessible format, please contact us at cmajgroup@cmaj.ca.

Prenatal	**Past history/ predisposing factors**	Not applicable
	**Asking about current anxiety, depression, and/or mood**	Not applicable
	**Use of EPDS**	Not applicable
	**Other Tool(s)**	Not applicable
Postnatal	**Past history/ predisposing factors**	Not applicable
	**Asking about current anxiety, depression, and/or mood**	Not applicable
	**Use of EPDS**	Not applicable
	**Other Tool(s)**	• During postnatal visit by a Community nurse, post-partum depression is one of the areas where community nurses can provide support[1].<br>• It is unclear whether any systematic or formal screening takes place.

[1] Whitehorse Health Center. Prenatal and post partum support. [Accessed 2020-02-17] Available: http://www.hss.gov.yk.ca/prenatalpostpartum.php

 Copyright © 2022 The Author(s) or their employer(s). To receive this resource in an accessible format, please contact us at cmajgroup@cmaj.ca.
