# Supplementary material for: Recommendation on instrument-based screening for depression during pregnancy and the postpartum period
Source: CMAJ. 2022 Jul 25;194(28):E981–9. doi: 10.1503/cmaj.220290 (PMC9328462; doi:10.1503/cmaj.220290)
Supplement: Appendix 5. Use of EPDS During Pregnancy and/or Postnatal Acros Provinces and Territories [file 220290-guide-5-at.pdf]

Appendix 5. Use of EPDS During Pregnancy and/or Postnatal Across Provinces and Territories

Province/Territory	*Type of Screening*	*Timing*	*Cut-offs*	
Pregnancy				
BC	Universal	28-32 weeks gestation[1]; rescreen in 2-4 weeks if EPDS score is 9-11	Score of 9-11: Depression possible. Support, rescreen in 2-4 weeks.<br>Score of 12-13: Fairly high possibility of depression: Monitor, support, offer education. Refer to primary care provider.<br>Score of 14 or above: Probable depression: diagnostic assessment and treatment.<br>Positive score on question 10 (suicidality risk): Immediate discussion and referral as required[2]	
AB				
SK	Universal	1st prenatal visit and at 28-34 weeks gestation[3,4]	Score of 10-11: Possible depression.<br>Score of 12 or above: probable depression. Offer referral to family doctor or nurse practitioner.<br>Positive score on question 10 (suicidality risk): Assess harm intentions and for psychosis[3]	
MB				
ON	Universal	2nd trimester[5]	Score of 10-12: monitor, support, and offer education<br>Score of 13 or above: follow up with comprehensive bio-psychosocial diagnostic assessment for depression<br>Positive score on question 10 (suicidality risk): immediate assessment and intervention[6]	Only as a second-line screening, if PHQ-2 score is positive; PHQ-9 may be administered instead of EPDS
QC	Opportunistic	Not provided	Score of 13 or above: refer for evaluation and treatment[7,8]	
NB	Universal	28-32 weeks gestation[9]	Score of 11-13: monitor, support, and offer education.<br>Score of 14 or above: follow-up with biopsychosocial diagnostic assessment for depression<br>Positive score on question 10 (suicidality risk): immediate assessment and intervention[9]	
NL				
NS				
PE	Opportunistic	Not provided	Not provided [10]	
NU	Universal	28-32 weeks[11,12]	Score of 14 or above OR<br>Positive score on question 10 (suicidality risk): refer to mental health services[12]	
NWT	Universal	28-32 weeks[13]	Score of 11-13: monitoring, support, education<br>Score of 14 or above: follow-up with biopsychosocial diagnostic assessment for depression<br>Positive score on question 10 (suicidality risk): immediate assessment and intervention[13]	
YK				
Postnatal				
BC	Universal	6-8 weeks post-partum[1]	Score of 9-11: Depression possible. Support, rescreen in 2-4 weeks.	

 Copyright © 2022 The Author(s) or their employer(s). To receive this resource in an accessible format, please contact us at cmajgroup@cmaj.ca.

			Score of 12-13: Fairly high possibility of depression: Monitor, support, offer education. Refer to primary care provider.<br>Score of 14 or above: Probable depression: diagnostic assessment and treatment.<br>Positive score on question 10 (suicidality risk): Immediate discussion and referral as required[2]	
AB	Universal	At first regular Public Health Well Child Clinic visit (generally at 2 months) [14]	Score of 10-12: Likelihood of depression considered moderate, discuss, inform of resources, follow-up based on clinical judgement.<br>Score of 12 or more: likelihood of depression is considered high, discuss, offer referral, develop a Family Support Plan, inform of services, follow-up based on clinical judgement.<br>Positive score on question 10 (suicidality risk): Use Suicide Risk flowchart, follow-up based on clinical judgement[15]	
SK	Universal	2-3 weeks postpartum, 2- or 4-month visit, and 6-month well child visits[4]	Score of 10-11: Possible depression: Probable depression:<br>Score of 12 or above: probable depression[3]<br>Positive score on question 10 (suicidality risk): Immediate discussion and referral required [4]	
MB				
ON	Universal	Unclear, in postnatal record form[6]	Score of 9-12: monitor, support, and offer education<br>Score of 13 or above: follow up with comprehensive bio-psychosocial diagnostic assessment for depression<br>Positive score on question 10 (suicidality risk): immediate assessment and intervention[6]	
QC	Unclear	Unclear, recommended in postnatal period[16]	Not provided[16]	
NB	Universal	6-8 weeks post-partum[9]	Score of 11-13: monitor, support, and offer education.<br>Score of 14 or above: follow-up with biopsychosocial diagnostic assessment for depression<br>Positive score on question 10 (suicidality risk): immediate assessment and intervention[9]	
NS	Universal	Not provided[17]	9 or above OR positive score on question 10 (suicidality risk): refer for follow-up[18]	
PE	Opportunistic	2 month child health clinic visit[10]	Nor provided	
NU	Universal	6-8 weeks post-partum[11]	Score of 14 or above OR<br>Positive score on question 10 (suicidality risk): refer to mental health services[12]	Differential guidance on cut-offs exists; federal direction specifies EPDS should be administered within 1 year post-partum and that cut-off score of 10 or more should be used[11]
NWT	Opportunistic	6 weeks post-partum[19]	Score of 11-13: monitoring, support, education	

 Copyright © 2022 The Author(s) or their employer(s). To receive this resource in an accessible format, please contact us at cmajgroup@cmaj.ca.

			Score of 14 or above: follow-up with biopsychosocial diagnostic assessment for depression<br>Positive score on question 10 (suicidality risk): immediate assessment and intervention[13]	
YK				

Note: Shaded cells indicates that no resources were available.

References for Appendix 5

1. Perinatal Services BC. (2012). Perinatal Services BC A Guide for Completion of the Antenatal Record Part 1 and 2. Retrieved February 24, 2020, from www.perinatalservicesbc.ca/Documents/Form/Form1582_Guide_AntenatalRecord1and2.pdf
2. BC Reproductive Mental Health Program and Perinatal Services BC. (2014). Edinburgh Perinatal/Postnatal Depression Scale (EPDS) Scoring Guide. Retrieved February 19, 2020, from http://www.perinatalservicesbc.ca/Documents/Resources/HealthPromotion/EPDS/EPDSScoringGuide_March2015.pdf
3. Saskatchewan Maternal Mental Health. (2020). Maternal Mental Health: EPDS Screening and Care Guide. Retrieved August 13, 2020 from: https://skmaternalmentalhealthca.files.wordpress.com/2018/09/edinburgh-postnatal-depression-screen-and-care-guide.pdf
4. Saskatchewan Prevention Institute. (2012). Edinburgh Postpartum Depression Scale (EPDS) Screening and Care Guide: Guide and Manual. Retrieved February 19, 2020, from https://skprevention.ca/resource-catalogue/mental-health/epds-screening-and-care-guide/
5. Provincial Council for Maternal and Child Health (PCMCH) and The Better Outcomes Registry & Network (BORN) Ontario Perinatal Record Working Group. (2018, August). A User Guide to the Ontario Perinatal Record. Retrieved February 19, 2020, from https://www.pcmch.on.ca/wp-content/uploads/2018/08/OPR_UserGuide_2018Update_Final_18-08-22.pdf
6. Ontario Ministry of Health and Long-Term Care. (2017). Ontario Perinatal Record. Retrieved August 13, 2020 from https://www.pcmch.on.ca/wp-content/uploads/2017/06/OPR-2017.pdf
7. Fournier, L., Roberge, P., Brouillet, H. (2012). Faire face à la dépression au Québec. Protocole de soins à l'intention des intervenants de première ligne. Montréal : Centre de recherche du CHUM. Retrieved February 24, 2020, from https://www.inspq.qc.ca/pdf/publications/1509_FaireFaceDepressionQc_ProtoSoinsInterv1reLigne.pdf
8. Institut national de santé publique du Québec. (2019). Santé mentale et troubles mentaux, Portail d'information périnatale. Retrieved February 24, 2020, from https://www.inspq.qc.ca/sites/default/files/documents/information-perinatale/sante-mentale.pdf
9. Horizon Health Network, & Vitality Health Network. (2015). The New-Brunswick Perinatal Health Program: A Completion Guide of the Antenatal Record. Retrieved February 19, 2020, from https://en.horizonnb.ca/media/755769/completion_guide_-_antenatal_record.pdf
10. Government of Prince Edward Island (2020). Pregnancy and Postpartum (Perinatal) Mood Disorders. Retrieved February 24, 2020, from https://www.princeedwardisland.ca/en/information/health-pei/pregnancy-and-postpartum-perinatal-mood-disorders
11. Maternal and Child Health Program, Department of Health and Social Services, Government of Nunavut. (2012, March). Retrieved February 25, 2020 from https://www.uvic.ca/medsci/assets/docs/arbour/Nunavut%20Prenatal%20Record%20Guidelines%20Version%202%20March%202012.pdf
12. Department of Health. (2016). Guidelines for Completing Prenatal Record. Retrieved February 19, 2020, from https://www.gov.nu.ca/sites/default/files/guidelines_for_completing_prenatal_record_april_2016_2.pdf
13. Government of Northwest Territories. (n.d.). NWT Prenatal Record – Part 1 to Part 6. Retrieved February 24, 2020, from https://www.hss.gov.nt.ca/professionals/sites/professionals/files/resources/nwt-prenatal-record.pdf
14. Maternal Newborn Child & Youth Strategic Clinical Network. (2019). Alberta Pregnancy Pathways. Retrieved February 24, 2020, from https://www.albertahealthservices.ca/assets/about/scn/ahs-scn-mncy-pp-nb-pathway.pdf
15. Alberta Health Services. (2019). Postpartum Depression Screening. Retrieved August 13, 2020 from: https://extranet.ahsnet.ca/teams/policydocuments/1/clp-prov-public-health-well-child-ppd-screen-guideline-hcs-229-01.pdf.
16. Fournier, L., Roberge, P., Brouillet, H. (2012). Faire face à la dépression au Québec. Protocole de soins à l'intention des intervenants de première ligne. Montréal : Centre de recherche du CHUM. Retrieved February 24, 2020, from https://www.inspq.qc.ca/pdf/publications/1509_FaireFaceDepressionQc_ProtoSoinsInterv1reLigne.pdf
17. Reproductive Care Program of Nova Scotia. (n.d.). Healthy Babies, Healthy Families: Postpartum & Postnatal Guidelines. Retrieved February 24, 2020 from http://rcp.nshealth.ca/sites/default/files/publications/healthy_babies_healthy_families.pdf

18. MacDonald, Joanne and Flynn, Coleen. (2012). Mothers' Mental Health Toolkit, A Resource for the Community. Retrieved February 24, 2020, from http://www.iwk.nshealth.ca/themes/iwkhc/downloads/mmh-toolkit.pdf
19. Government of Northwest Territories. (2017). A User's Guide for Completion of the NWT Prenatal Record, 2017. Retrieved February 19, 2020, from https://www.hss.gov.nt.ca/professionals/sites/professionals/files/resources/prenatal-record-users-guide.pdf

Appendix 5, as supplied by the authors. Appendix to: Lang E, Colquhoun H, LeBlanc JC, et al; Canadian Task Force on Preventive Health Care Recommendation on instrument-based screening for depression during pregnancy and the postpartum period. *CMAJ* 2022. doi: 10.1503/cmaj.220290. Copyright © 2022 The Author(s) or their employer(s). To receive this resource in an accessible format, please contact us at cmajgroup@cmaj.ca.
